# Transcriptome-Based Network Analysis Unveils Eight Immune-Related Genes as Molecular Signatures in the Immunomodulatory Subtype of Triple-Negative Breast Cancer

**DOI:** 10.3389/fonc.2020.01787

**Published:** 2020-09-18

**Authors:** Jinguo Zhang, Li Wang, Xiaolin Xu, Xin Li, Wencai Guan, Ting Meng, Guoxiong Xu

**Affiliations:** ^1^Research Center for Clinical Medicine, Jinshan Hospital, Fudan University, Shanghai, China; ^2^Department of Oncology, Shanghai Medical College, Fudan University, Shanghai, China; ^3^Department of Pathology, Jinshan Hospital, Fudan University, Shanghai, China; ^4^Center for Tumor Diagnosis and Therapy, Jinshan Hospital, Fudan University, Shanghai, China

**Keywords:** breast cancer, gene signature, immunomodulatory subtype, overall survival, transcriptome-based network, WGCNA

## Abstract

**Objective:** Triple-negative breast cancer (TNBC) is a high heterogeneity cancer. The identification of genomic aberrations that drive each of the TNBC subtypes may predict the prognosis of patients with TNBC and provide novel therapeutic strategies in clinical practice. This study focuses on the transcriptome-based gene expression of TNBC and aims to generate comprehensive gene co-expression networks correlated with the immune-related subtype of TNBC.

**Methods:** The transcriptome profiles of 107 female patients with TNBC were analyzed. Weighted gene co-expression network analysis (WGCNA) was applied to construct related networks and to sort hub-genes associated with the survival of TNBC patients. The data of the transcriptional expression, genomic alteration, survival status, and tumor immune microenvironment, which associated with hub-genes, were extracted, retrieved, and analyzed from Oncomine, UALCAN, TCGA, starBase, Kaplan–Meier Plotter, cBioPortal, and TIMER databases.

**Results:** Immune-related hub-genes, including *BIRC3, BTN3A1, CSF2RB, GIMAP7, GZMB, HCLS1, LCP2*, and *SELL*, were found to be associated with clinical features of TNBC evaluated by WGCNA. These hub-genes belonged to the immunomodulatory subtype of TNBC and were upregulated in the TNBC cells. The protein expression of eight immune-related hub-genes was further confirmed to be upregulated in TNBC/CD8+ tissues detected by immunohistochemical staining. Survival analysis revealed that overexpression of eight immune-related hub-genes was in favor of the survival of patients with TNBC. Moreover, a positive correlation between eight immune-related hub-genes and immune cell infiltration was observed in TNBC patients. Furthermore, checkpoint inhibitor genes such as *PD-L1, PD-1*, and *CTLA4* were more enrichment in the immunomodulatory subtype of TNBC and the expression of PD-L1, PD-1, and CTLA4 was positively correlated with eight immune-related hub-genes in the breast cancer dataset of TCGA.

**Conclusions:** Eight immune-related hub-genes were identified to be molecular signatures in the immunomodulatory subtype of TNBC, which may provide therapeutic targets for the treatment of patients with breast cancer.

## Introduction

Breast cancer is the most common malignant tumor in women (1). The intrinsic subtypes of breast cancer have been elucidated with advances in cancer genomics, which lead to the generalization of therapies by targeting human epidermal growth factor receptor 2 (HER2) for HER2-positive patients and endocrine therapy for hormone receptor-positive patients (2, 3). Triple-negative breast cancer (TNBC) is a subtype of breast cancer defined by the lack of the expression of estrogen receptor (ER), progesterone receptor (PR), and HER2 (4). TNBC accounts for ~15–20% of all breast cancer cases with a relatively poor outcome compared to other breast cancer subtypes (5). The main difficulty for the management of patients with TNBC is that there is greater heterogeneity of clinical behavior and the absence of recognized molecular targets for treatment (6). Generally, breast cancer metastasis is one of the most important causes of treatment failure, which exerts a dramatically negative impact on the cure and survival of patients (7, 8). Moreover, patients with TNBC are associated with an increase in distant metastasis and shorter metastasis-free survival after chemotherapy (9, 10). It has been shown that the incidence of visceral metastasis in patients with TNBC is higher than non-TNBC patients (11). Therefore, the mechanism underlying TNBC progression and metastasis is needed to explore and novel therapeutic approaches are needed to define.

In recent years, TNBC has clinically been sub-divided into six molecular subtypes based on multi-omics and sequencing techniques: basal-like 1 (BL1), basal-like 2 (BL2), mesenchymal (M), mesenchymal stem-like (MSL), immunomodulatory (IM), and luminal androgen receptor (LAR) (12, 13). More recently, carcinogenic mutations and biomarker expression in TNBC are widely examined and reported (14). Prognostic and predictive biomarkers may provide important tools for the precision medicine of individual TNBC therapy. However, the potentially driving molecular events within each TNBC subtype are poorly understood. It seems that most studies have focused on single molecules excessively, while their interactors among molecules are largely neglected. Therefore, more extensive genomic, expression profile analyses of TNBCs are needed to elucidate the complexity of the disease and to identify appropriate biomarkers for distinguishing different TNBC subtypes.

Weighted gene co-expression network analysis (WGCNA) has been emerged as an effective biology algorithm to construct a co-expression network across gene expression data, exploring the association between gene networks and phenotypes of interest (15). The highly related genes may present an expression pattern that shares common biological functions (16). The hub-genes in the cluster defined by WGCNA may be candidate biomarkers or therapeutic targets for the disease (17) and the mechanism of breast cancer progression would be elucidated by the regulatory networks of related molecules (18). Furthermore, WGCNA may identify prognostic biomarkers associated with ER-positive breast cancer and poor survival (19) and be used to evaluate the association between gene co-expression clusters and responses to neoadjuvant chemotherapy in a large-scale breast cancer dataset (20). However, less is known about the study of gene co-expression analyses on the immunomodulatory module of TNBC.

The present study focused on the transcriptome-based gene expression of TNBC and aimed to generate comprehensive gene co-expression networks correlated with the immune-related subtype of TNBC. The association of TNBC with survival and metastasis was evaluated in patients with breast cancer. Several immune-related key genes at the levels of mRNA, protein, genomic alteration frequency, and tumor immune microenvironment were also analyzed and validated.

## Materials and Methods

### Public Datasets and Data Preprocessing

The data were retrieved from the Gene Expression Omnibus database (GEO, https://www.ncbi.nlm.nih.gov/geo/). The mRNA expression profiles were obtained from the dataset with the accession number GSE58812 (GEO platform GPL570), which contains 107 TNBC surgical specimens for conducting the primary analysis (21). Another GEO dataset GSE22133 (GEO platform GPL4723) containing 78 TNBC patients was used for validation (22). Both GEO datasets provided information about survival status and metastasis. None of the patients received chemotherapy, radiotherapy, or endocrine therapy before surgery. Probes without gene annotation or probes matched more than one gene symbols were excluded. The top 3,600 genes in 107 samples from GEO were selected to construct the co-expression networks after sorting their variances ranked in descending order.

### Co-expression Module Detection

WGCNA was performed on the software R (version 3.4.0) with the “wgcna” R package (17). Generally, the topology of the co-expression network was constructed based on the scale-free network. The soft-threshold power β was selected by the function of softConnectivity from package WGCNA, which may influence the scale independence and mean connectivity of the network (15). When the scale-free Topology Fit Index (TFI) reaches a value above 0.9 for low powers (<30), the topology of the gene coexpression network is scale-free and no batch-effects (23). Based on the mRNA expression data of TNBC, an adjacency matrix was computed and then transformed into a topological overlap matrix (TOM) with a standard procedure of WGCNA (15). Next, the hybrid dynamic tree-cutting algorithm was applied to identify network modules using the TOM dissimilarity (24). The minimum module size and the medium sensitivity were set as 30 and 2, respectively. Other parameters were set as default. Different colors indicated different modules. The gray module referred to a gene group that could not be classified into any modules. Module eigengenes (MEs) were computed by retaining the first principal component that represented a module. Correlations between module eigengenes and clinical traits (survival status and metastasis) were calculated to identify a module that highly related to survival and metastasis. Gene significance (the correlation between the genes and the clinical traits) and module membership (the correlation between each module eigengene and the expression profile) were also calculated.

### Functional Annotation of Co-expression Modules

The interested module related to survival and metastasis was selected. These genes in a selected module were used to conduct functional enrichment analysis. The Gene Ontology (GO) and Kyoto Encyclopedia of Genes and Genomes (KEGG) pathway enrichment analyses were performed with the DAVID database (https://david.ncifcrf.gov/) (25). If there were more than 10 GO annotation and pathway enrichments, only the top 10 terms with a *P* < 0.05 were extracted. Furthermore, the Metascape tool (http://metascape.org) was applied for functional annotation of immune-related genes and its closely related neighbor genes (26). The top 10 enriched biological processes and pathways were also analyzed.

### Detection of Hub-Genes and Construction of Protein-Protein Interaction (PPI) Network

The hub-genes are defined as a series of genes that are the most connected and main core in a module (27). A network of screening function based on gene significance and module membership was used to screen hub-genes as described previously (17, 28). The top 50 hub-genes with the cut-off criteria of *q*-weighted value < 0.001 were detected. The PPI network of hub-genes was constructed by the Search Tool for the Retrieval of Interacting Genes (STRING) database (https://string-db.org/) (29).

### Survival Analysis of Hub-Genes

In the discovery dataset (GEO: GSE58812), the raw clinical data including survival status and time were extracted. A univariate Cox proportional hazards regression model was used to regress patient overall survival by using the median expression level of hub-genes. All results were processed by SPSS (SPSS24.0, Inc. Chicago, IL, USA). In the validation dataset (GEO: GSE22133), a comprehensive survival analysis tool PROGgeneV2 (www.compbio.iupui.edu/proggene) was applied to conduct a log-rank survival analysis on the primary endpoint of overall survival based on the median expression value of hub-genes (30).

### Oncomine Analysis

The gene expression array datasets were extracted from a publicly accessible, web-based cancer microarray database Oncomine (www.oncomine.org). In the present study, the mRNA expression levels of immune-related genes in different breast cancer tissues and their corresponding adjacent normal control samples were analyzed. The thresholds were determined as follows: *P* = 0.05; fold-change = 1.5, mRNA data type, and 10% gene ranking.

### The Cancer Genome Atlas (TCGA) Data Mining

UALCAN (http://ualcan.path.uab.edu) was a comprehensive web resource, which provided RNA-seq and clinical data of 31 cancer types based on TCGA (31). In this study, UALCAN was applied to analyze the expression of immune-related genes in TNBC and normal breast tissues in each TNBC subtype. Furthermore, the association between hub-gene expression and nodal metastasis status was also analyzed. For exploring the relationship between those immune-related genes and immune checkpoint inhibitor genes, the statBase online tool (http://starbase.sysu.edu.cn/panCancer.php) was used in the Pan-Cancer analysis platform for TCGA-BRCA data (32).

### Survival Analysis in Kaplan–Meier Plotter

The Kaplan–Meier plotter (www.kmplot.com), a web-based tool containing gene expression data and the survival information of patients from GEO and TCGA (33), was used to assess the survival of TNBC patients associated with the mRNA expression of immune-related genes. The overall survival of 1,402 breast cancer patients and relapse-free survival of 255 TNBC patients were plotted with an auto-selected best cut-off value (high *vs*. low expression). The hazard ratio (HR) with 95% confidence intervals (CIs) and log-rank *P*-value were also calculated.

### cBioPortal Database Analysis

cBioPortal for Cancer Genomics (www.cbioportal.org), an online open-access website resource, was used to explore, visualize, and analyze multidimensional cancer genomics data (34). In this study, we analyzed the genomic profiles of eight immune-related genes, which contained mutations, putative copy-number alterations, and mRNA expression. In cBioPortal, a TCGA PanCancer Atlas dataset with 994 complete samples of breast invasive carcinoma was chosen. Next, genomic profiles of mutations, putative copy-number alterations, and mRNA expression were selected, mRNA expression z scores (RNA Seq V2 RSEM) were obtained with a z score threshold of ±2.0. The OncoPrint presented an overview of genetic alterations per sample in those immune-related genes. The alteration frequency derived from mutations, copy-number alterations, and mRNA expression data was analyzed in breast invasive carcinomas. The association of genetic alterations in eight immune-related hub-genes with the subtype of breast cancer, mutation frequency, overall survival, disease-free survival, progression-free survival, and disease-specific survival of breast cancer patients was computed. The log-rank test was performed to identify the significant difference in the survival curves.

### TIMER Database Analysis

The Tumor IMmune Estimation Resource (TIMER) database (https://cistrome.shinyapps.io/timer/) is a reliable and comprehensive resource that allows the evaluation of the abundance of immune cell infiltration across diverse cancer types (35). In this study, the “Gene module” was used to evaluate the correlation between the expression level of immune-related genes, tumor purity, and the infiltration of immune cells including B cells, CD4^+^ T cells, CD8^+^ T cells, neutrophils, macrophages, and dendritic cells (DCs) in basal-like breast cancer. Meanwhile, the “Correlation module” was used to investigate the correlation between the expression level of immune-related genes and several gene markers of tumor-infiltrating immune cells.

### Detection of the Expression of Hub-Genes by qRT-PCR

Total RNA was extracted from the luminal breast cancer cell line MCF-7, and TNBC cell lines MDA-MB-231 and MDA-MB-468 using the RNA-Quick Purification Kit (Yishan Biotechnology Co., Ltd., Shanghai, China). RNA quantity and quality were measured and assessed by A260/A280 absorption. Total RNA was reverse transcribed using a Transcriptor First Strand cDNA Synthesis Kit (Roche Applied Science, Switzerland). The FastStart Universal SYBR-Green Master kit (Roche Applied Science) was used to perform PCR at the following conditions: 95°C for 10 min, followed by 40 cycles of 95°C for 10 s, and 60°C for 30 s. The primer sequences of hub-genes and GAPDH were shown in Supplementary Table 1. The relative expression level of target genes was calculated using the 2^−ΔΔCT^ method.

### Immunohistochemistry

The study of the human subject was approved by the Ethics Committee of Jinshan Hospital, Fudan University. Ten breast cancer tissues were obtained from Jinshan Hospital, including five TNBC and five non-TNBC biopsy specimens, respectively. All tissues underwent pathological examination after surgery in the Department of Pathology, Jinshan Hospital. After paraformaldehyde-fixed paraffin-embedded tissues were sectioned, deparaffinized, and dehydrated, the sections were stained with different antibodies obtained from ProteinTech Group (Chicago, IL, USA). The following antibodies were used: anti-BIRC3 (#24304-1-AP), anti-BTN3A1 (#25221-1-AP), anti-CSF2RB (#27148-1-AP), anti-GZMB (#13588-1-AP), anti-GIMAP7 (#17293-1-AP), anti-HCLS1 (#25003-1-AP), anti-LCP2 antibody (#12728-1-AP), and anti-SELL (#26477-1-AP).

### Statistical Analysis

For WGCNA network construction, all plots were generated using software R (version 3.5.1). Survival Analysis of hub-genes was processed by SPSS. Differential expression of eight immune-related genes between the two groups was compared by a two-tailed Student's *t*-test. Data were presented as mean ± the standard deviation. Statistical analysis was performed using GraphPad Prism 8.0 (GraphPad Software Inc., San Diego, CA, USA). A *P* < 0.05 was considered statistically significant.

## Results

### Construction of Co-expression Modules and Module-Trait Relationship of TNBC

**Figure 1:**
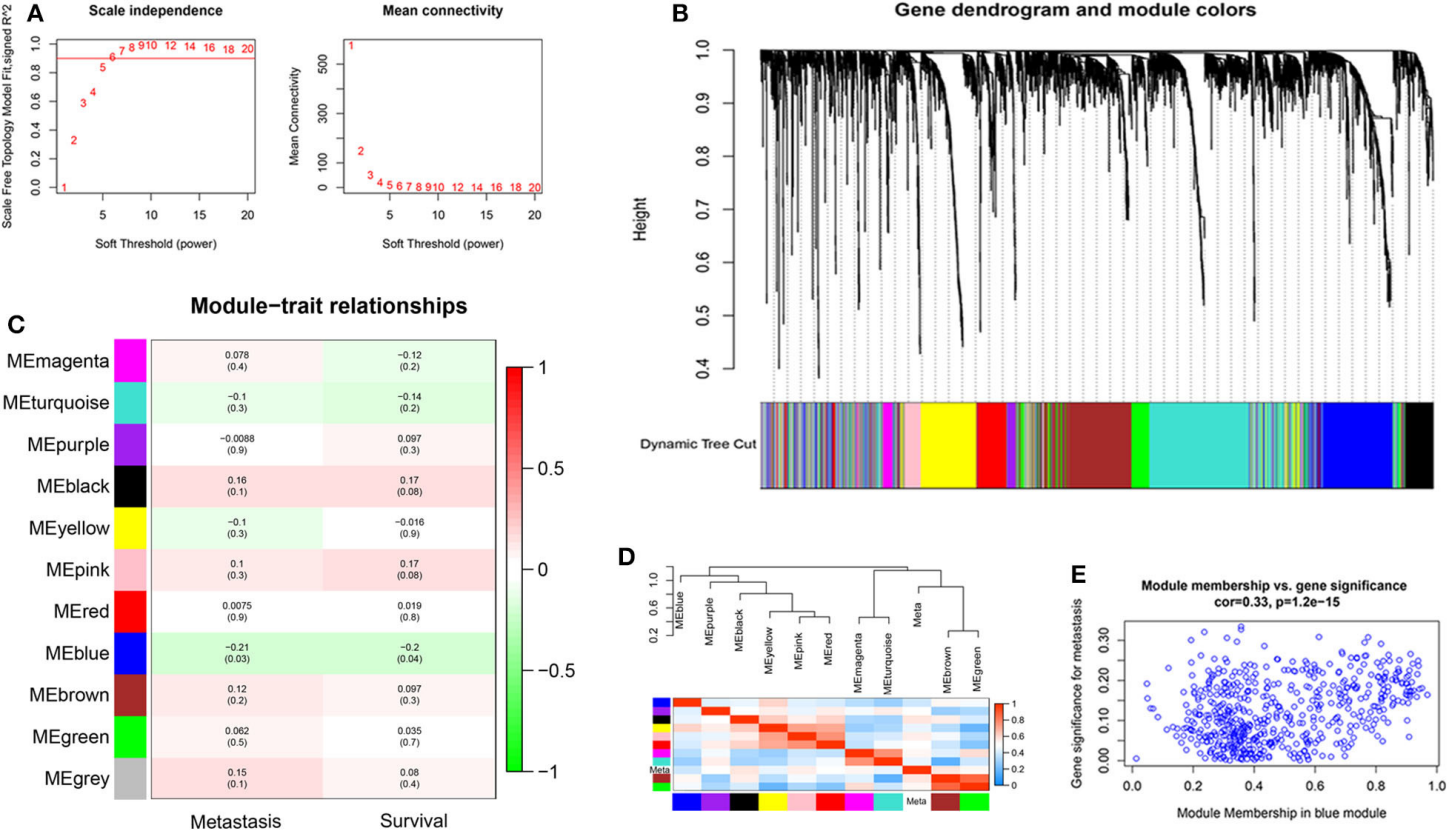
The construction of co-expression modules and module-trait relationships of TNBC. **(A)** Analysis of network topology for different soft-thresholding powers. The left panel shows the influence of soft-thresholding power (*x*-axis) on the scale-free fit index (*y*-axis). The right panel displays the influence of soft-thresholding power (*x*-axis) on the mean connectivity (degree, *y*-axis). **(B)** Clustering dendrogram of 3,600 genes from the dataset GSE58812 with 105 TNBC surgical specimens. A hierarchical cluster analysis was applied to detect co-expression clusters with dissimilarity based on the topological overlap. Each short vertical line corresponds to a gene. The branches are modules of highly interconnected groups of gene expression. Different modules were identified and shown in different colors. **(C)** Analysis of module-trait relationships of TNBC based on the dataset GSE58812. **(D)** Visualizing the gene network using a heatmap plot. **(E)** Scatter plot of Gene Significance (GS) vs. Module Membership (MM) in the co-expression blue module.

Probes with variances ranked in the top 3,600 genes in 107 samples of TNBC were applied to construct a co-expression module for the subsequent analyses. Because of the presence of outliers (Supplementary Figure 1), two samples were removed for further analysis. The soft-threshold was set up by the softConnectivity function. When the power value reaches six, the scale-free topology fit index was up to 0.9. Thus, we selected power value 6 to calculate the adjacency matrix and to construct a co-expression module to see the influence of soft-thresholding power on the scale-free fit index and the mean connectivity (Figure 1A). A hierarchical cluster analysis of 3,600 genes from the dataset GSE58812 in 105 TNBC surgical specimens was applied to detect co-expression clusters with dissimilarity based on the topological overlap. We found that 11 co-expression modules by the dynamic tree-cutting methods (Figure 1B). These modules displayed in different colors were clustered from all genes ranged from 69 to 838 in size (Supplementary Table 2). The clinical data of 105 patients were obtained from GEO, including age, overall survival, and metastasis status. The module-trait association was evaluated using the correlation between the module eigengene and the clinical features such as survival and metastasis status. Interestingly, we found a negative correlation between the blue module and these clinical features with a *P* < 0.05 (Figure 1C). Next, the module eigengenes dendrogram and heatmap also confirmed that the blue module significantly had a negative correlation with TNBC survival and metastasis (Figure 1D). Finally, a scatterplot of Gene Significance (GS) vs. Module Membership (MM) was applied for the co-expression blue module (Figure 1E).

### Functional Annotation of the Blue Module and Eight Immune-Related Genes Screening

**Figure 2:**
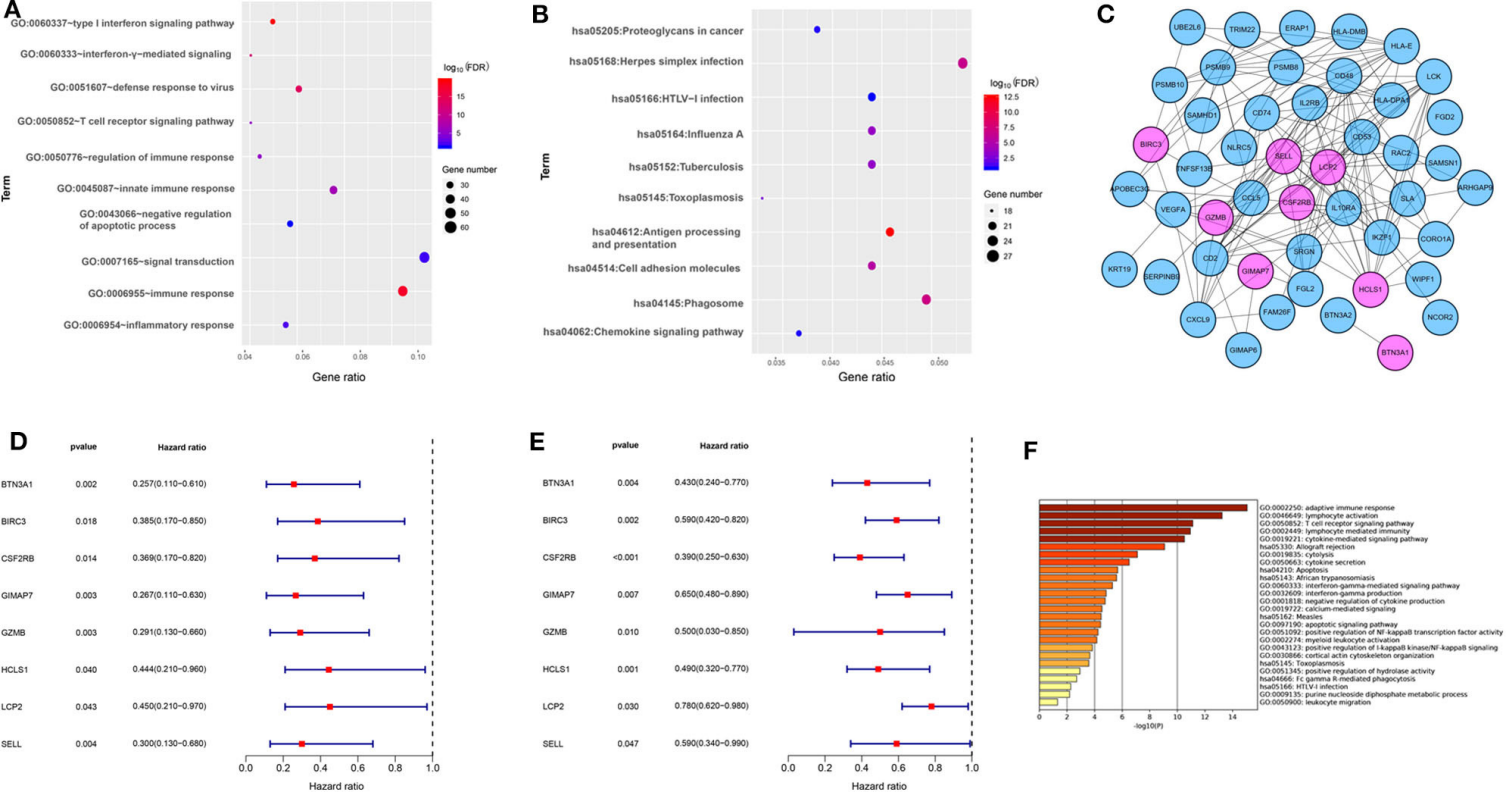
The functional annotation of the blue module and eight immune-related genes screening. **(A)** The enrichment analysis of the blue module in biological process terms (DAVID 6.8). **(B)** The enrichment analysis of the blue module in KEGG enriched terms. **(C)** Protein-protein interaction network of 50 hub-genes based on the STRING database. The nodes stand for the hub-genes and the lines represent interactions between hub-genes. The pink nodes represent the eight immune-related hub-genes that were associated with the survival of TNBC patients. **(D)** Relationships of eight immune-related hub-genes with overall survival in the training dataset GSE58812. **(E)** Relationships of eight immune-related hub-genes with overall survival in the validation dataset GSE22133. **(F)** The enrichment analysis of eight immune-related hub genes and 80 most frequently altered neighboring genes in breast cancer (Metascape).

To better understand the biological function of the blue module, the GO enrichment, and KEGG pathways of the blue module were analyzed. Figures 2A,B showed the top 10 GO terms and the top 10 KEGG pathways, respectively. Using the network screening function method, the top 50 hub-genes were identified to relate to the metastasis and survival of TNBC patients based on the GS and MM. The top 50 hub-genes in the blue module were listed in Supplementary Table 3. Using the STRING database, a PPI network with 49 nodes and 181 edges was constructed (Figure 2C).

To explore the potential prognostic value, the top 50 hub-genes were subjected to survival analysis in the training and validation datasets. We found that lower expressions of eight hub-genes (*BTN3A1, BIRC3, CSF2RB, GIMAP7, GZMB, HCLS1, LCP2*, and *SELL*) were significantly correlated with worse overall survival of patients with TNBC in the training (Figure 2D) and the validation datasets (Figure 2E). To elucidate the biological function of eight hub-genes and their related neighboring genes, we used the Metascape tool to conduct the GO term and pathway analyses. The functions of eight hub-genes and their neighboring genes were mainly enriched in adaptive immune response and cytokine-mediated signaling pathways (Figure 2F), suggesting that eight hub-genes may mainly be involved in immune processes.

### Aberrant Expression of Eight Immune-Related Genes in TNBC Patients

**Figure 3:**
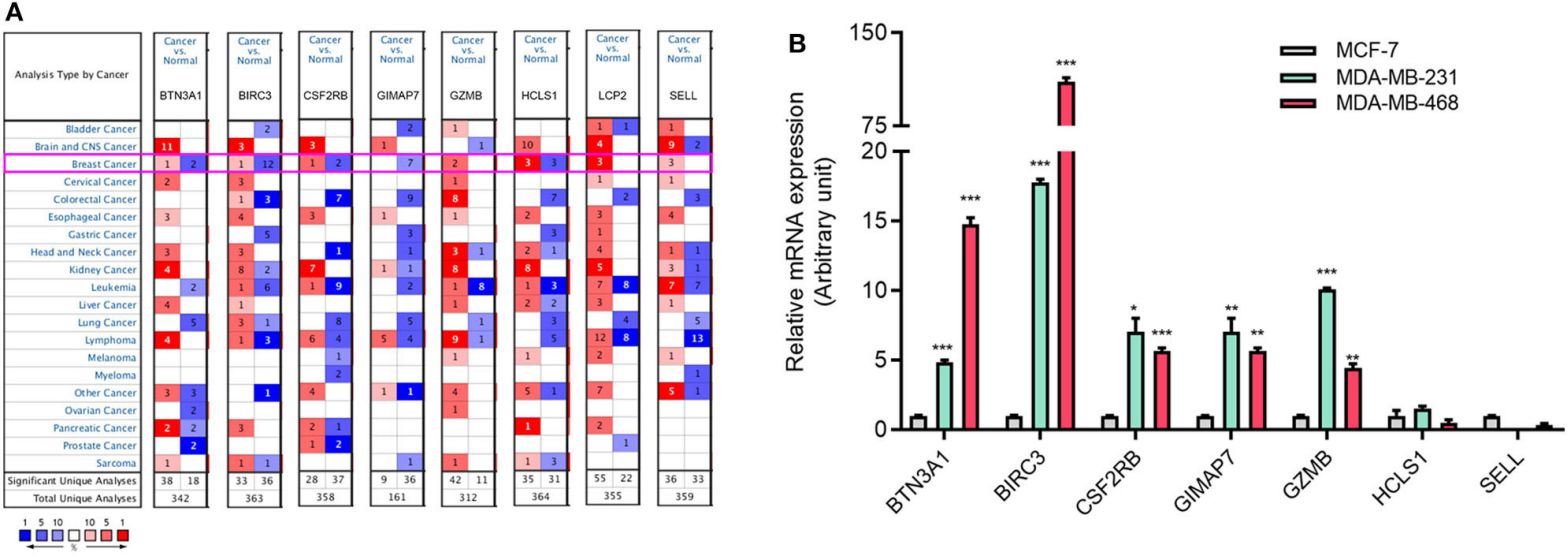
The aberrant expression of eight immune-related hub-genes in TNBC patients. **(A)** The mRNA levels of eight immune-related hub-genes in breast cancer (Oncomine). The number in each cell represents the number of datasets with statistically significant mRNA overexpression (red) or downexpression (blue) of target genes. **(B)** The mRNA levels of seven immune-related genes in TNBC cell lines MDA-MB-231 and MDA-MB-468 cells compared with luminal breast cancer cell line MCF-7 cells by qRT-PCR. *p < 0.05, **p < 0.01, ***p < 0.001.

To investigate the role of eight immune-related genes in TNBC, we conducted compressive analyses of mRNA, genomic alteration, and tumor immune microenvironment in TNBC. First, we explored the transcriptional level of eight immune-related genes in breast cancer and normal breast tissues based on the Oncomine database. The transcriptional levels of GZMB, LCP2, and SELL were significantly upregulated (Table 1), whereas GIMAP7 was significantly downregulated, in breast cancer compared with normal breast tissue. However, the mRNA levels of BTN3A1, BIRC3, CSF2RB, and HCLS1 were controversial (Figure 3A). In the Curtis dataset (36), the expression of GZMB was 5.918 times higher in medullary breast carcinoma tissue than normal tissue. In the Finak dataset (37), the expression of GZMB was 4.910 times higher in invasive breast carcinoma than normal tissue. LCP2 expression was elevated in breast cancer compared with normal breast tissues in three datasets. The transcriptional level of LCP2 was higher in invasive ductal breast carcinoma than normal breast tissues in the Karnoub dataset (*p* = 2.52e-6) and the Zhao dataset (*p* = 3.82e-5) (38, 39). Similarly, LCP2 was significantly upregulated in ductal breast carcinoma *in situ* in the Ma dataset (40). The fold change of LCP2 expression in breast cancer was 3.240, 1.543, and 2.166, in the datasets of Karnoub, Zhao, and Ma, respectively. Furthermore, overexpression of SELL mRNA was found in breast cancer tissues in the datasets of Curtis, TCGA, and Ma with the fold change of 2.687, 2.525, and 2.614, respectively (36, 40).

**Table 1:** Significant changes of three hub-genes at the transcriptional level between breast cancer and normal breast tissues.

**Gene name**	**Type of breast cancer**	**FC**	***P*-value**	***t*-test**	**Source/Reference**
GZMB	Medullary Breast Carcinoma	5.918	1.37E-11	9.796	ONCOMINE (36)
	Invasive Breast Carcinoma	4.910	4.20E-12	10.072	ONCOMINE (37)
LCP2	Invasive Ductal Breast Carcinoma	3.240	2.52E-6	6.876	ONCOMINE (38)
	Ductal Breast Carcinoma *in situ*	2.166	6.44E-4	4.179	ONCOMINE (40)
	Invasive Ductal Breast Carcinoma	1.543	3.82E-5	5.301	ONCOMINE (39)
SELL	Medullary Breast Carcinoma	2.687	3.62E-8	6.911	ONCOMINE (36)
	Invasive Breast Carcinoma	2.525	1.16E-12	7.733	TCGA Breast
	Ductal Breast Carcinoma *in situ*	2.614	0.006	2.996	ONCOMINE (40)

*P-value from the comparison of breast cancer vs. normal breast tissue. FC, fold change; GZMB, Granzyme B; LCP2, lymphocyte cytosolic protein 2; SELL, selectin L*.

### Aberrant Expression of Immune-Related Genes in TNBC and the Subtype of TNBC

To explore the expression of eight immune-related genes in TNBC cell lines, we performed qRT-PCR in TNBC cell lines MDA-MB-231 and MDA-MB-468 compared with luminal breast cancer cell line MCF-7. The expression of five immune-related genes (BTN3A1, BIRC3, CSF2RB, GIMAP7, and GZMB) was higher in MDA-MB-231 and MDA-MB-468 cells than MCF-7 cells (Figure 3B). The expression of LCP2 was not detectable in MCF-7 and MDA-MB-231 cells but was detectable in MDA-MB-468 cells (data not shown).

**Figure 4:**
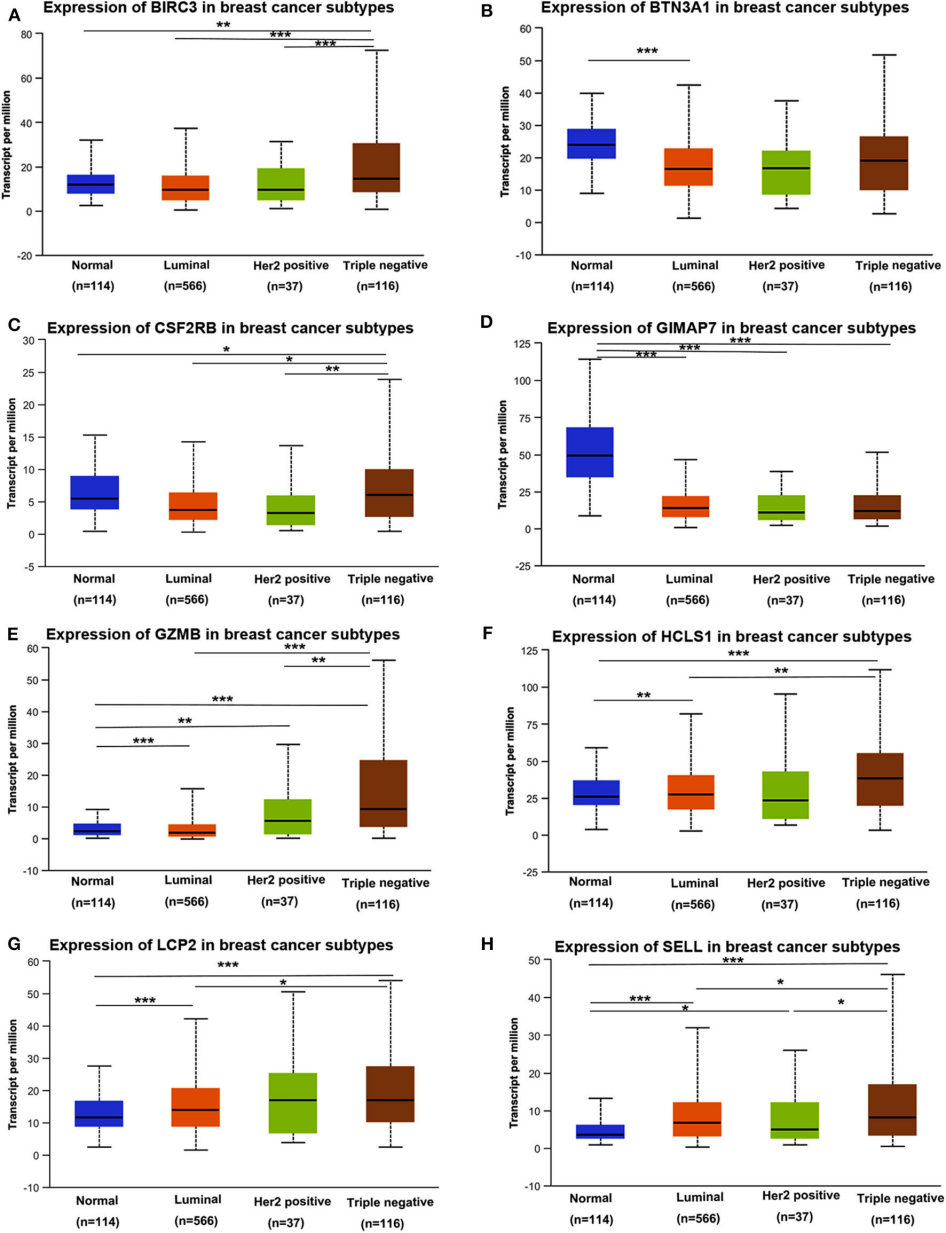
The transcription level of eight immune-related hub-genes in breast cancer subtypes (UALCAN). The transcriptional levels of BIRC3 **(A)**, BTN3A1 **(B)**, CSF2RB **(C)**, GIMAP7 **(D)**, GZMB **(E)**, HCLS1 **(F)**, LCP2 **(G)**, and SELL **(H)** are shown. *p < 0.05, **p < 0.01, ***p < 0.001.

Next, we assessed the expression levels of immune-related genes in TCGA cohorts and normal tissues with UALCAN. We found that the mRNA levels of BIRC3 (*p* = 2.16e−3), CSF2RB (*p* = 3.27e−2), GZMB (*p* = 2.15e−10), HCLS1 (*p* = 5.85e−5), LCP2 (*p* = 7.27e−7), and SELL (*p* = 3.18e−6) were significantly elevated in TNBC compared with normal breast tissues, while the transcriptional levels of GIMAP7 (*p* = 1.62e−12) were significantly reduced (Figure 4). The TNBC group was also compared to luminal and Her2-positive groups. The expression of BIRC3, CSF2RB, G2MB, and SELL was higher in the TNBC group than Luminal and HER2-positive groups, whereas the expression of HCLS1 and LCP2 was higher in the TNBC group than the luminal group.

**Figure 5:**
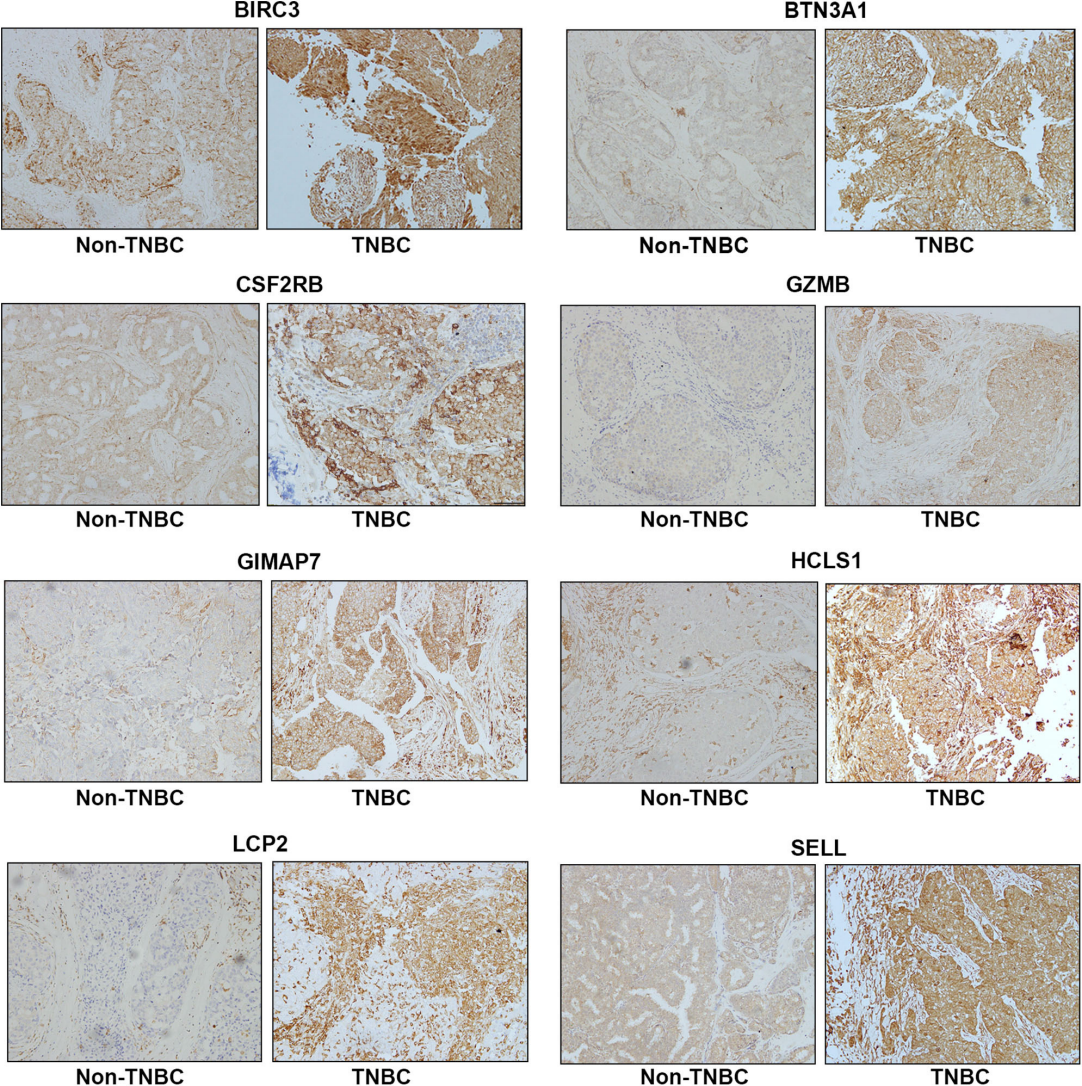
Representative immunohistochemistry staining for eight immune-related protein expression in Non-TNBC and TNBC tissues. Magnification, ×200.

To assess the protein expression of eight immune-related genes in TNBC vs. non-TNBC, we performed immunohistochemical staining. The expression levels of eight proteins were higher in TNBC tissues than non-TNBC tissues (Figure 5).

We then evaluated the correlation between the expression of eight immune-related genes and the nodal metastasis status of breast cancer patients. A significant correlation between the expression of these genes (BIRC3, GZMB, HCLS1, LCP2, and SELL) and nodal metastasis status was detected (Supplementary Figure 2).

**Figure 6:**
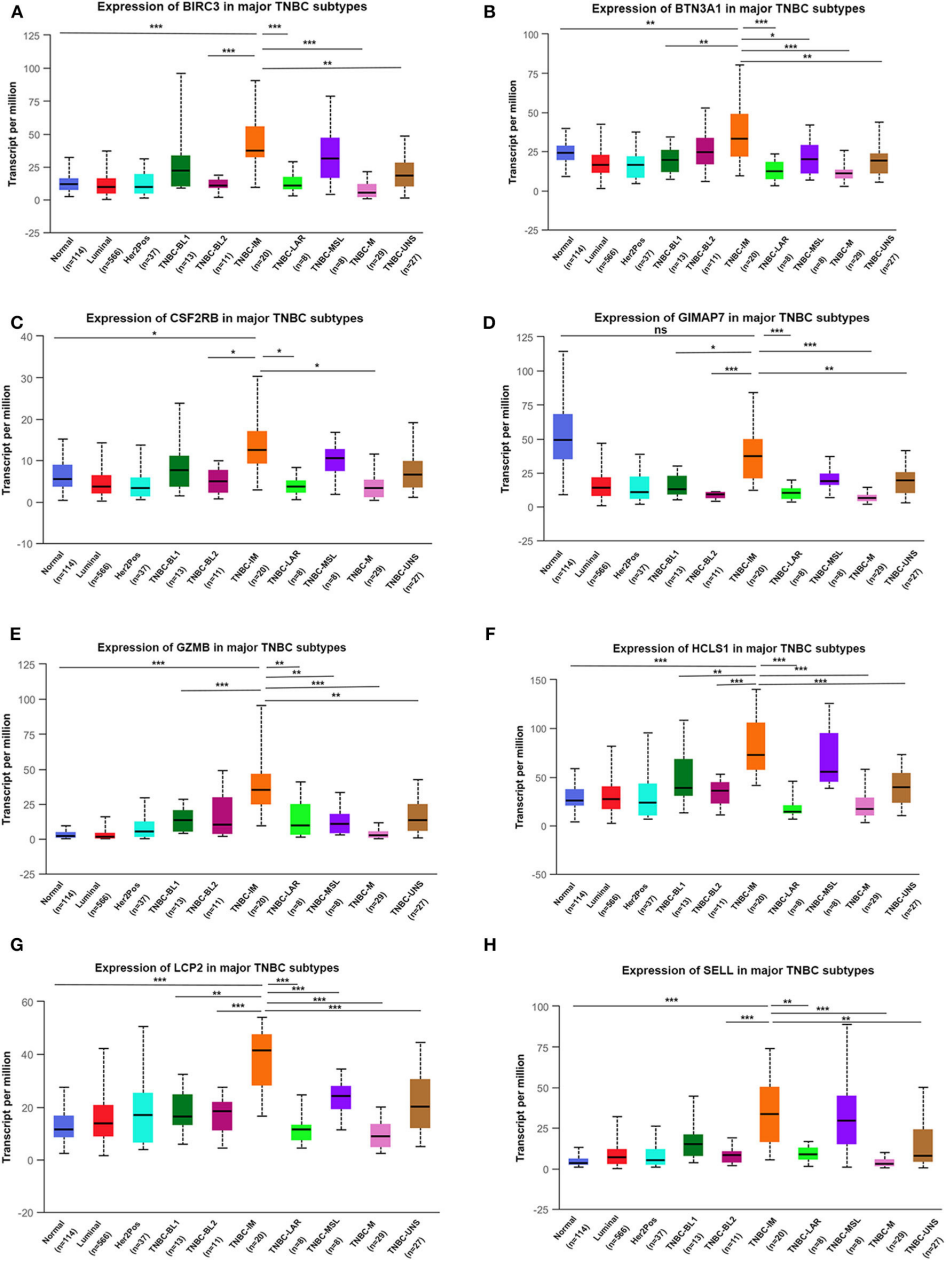
The transcription level of eight immune-related genes in TNBC subtypes (UALCAN). The transcriptional levels of BIRC3 **(A)**, BTN3A1 **(B)**, CSF2RB **(C)**, GIMAP7 **(D)**, GZMB **(E)**, HCLS1 **(F)**, LCP2 **(G)**, and SELL **(H)** are shown. *p < 0.05, **p < 0.01, ***p < 0.001; ns, no significance. BL1, basal-like 1; BL2, basal-like 2; IM, immunomodulatory; M, mesenchymal; MSL, mesenchymal stem-like; LAR, luminal androgen receptor; UNS, unspecified; TNBC, triple-negative breast cancer.

TNBC is a highly diverse group of cancer and can be classified into six molecular subtypes (12). To investigate the expression of eight immune-related genes in the TNBC subtypes, we also analyzed the transcriptomic data of eight immune-related genes in the TNBC subtype of the TCGA database with UALCAN. Intriguingly, the expression levels of BIRC3, BTN3A1, CSF2RB, GZMB, HCLS1, LCP2, and SELL were dramatically increased in TNBC immunomodulatory (IM) subtype (tumors *vs*. normal breast tissues). Furthermore, significantly higher mRNA expressions of eight immune-related genes were observed in the immunomodulatory subtype of TNBC compared with other TNBC subtypes (Figure 6).

Next, we examined the association of the expression of eight immune-related genes with age (younger vs. older), stage (early vs. later), and menopausal status (pre-menopause vs. post-menopause) in patients with breast cancer. The level of BIRC3 expression was significantly higher in the 41–60 years group than the 61–80 years group and the level of SELL expression was significantly higher in the 21–40 years group than the 61–80 years group (Supplementary Figure 3). The level of BIRC3 expression was significantly higher in the stage 1–2 groups than the stage 3 group and the lowest level of GZMB expression was found in the stage 1 group (Supplementary Figure 4). However, the expression of eight immune-related genes was not associated with menopause (Supplementary Figure 5).

### The Prognostic Value of Eight Immune-Related Genes in Patients With Breast Cancer and TNBC

**Figure 7:**
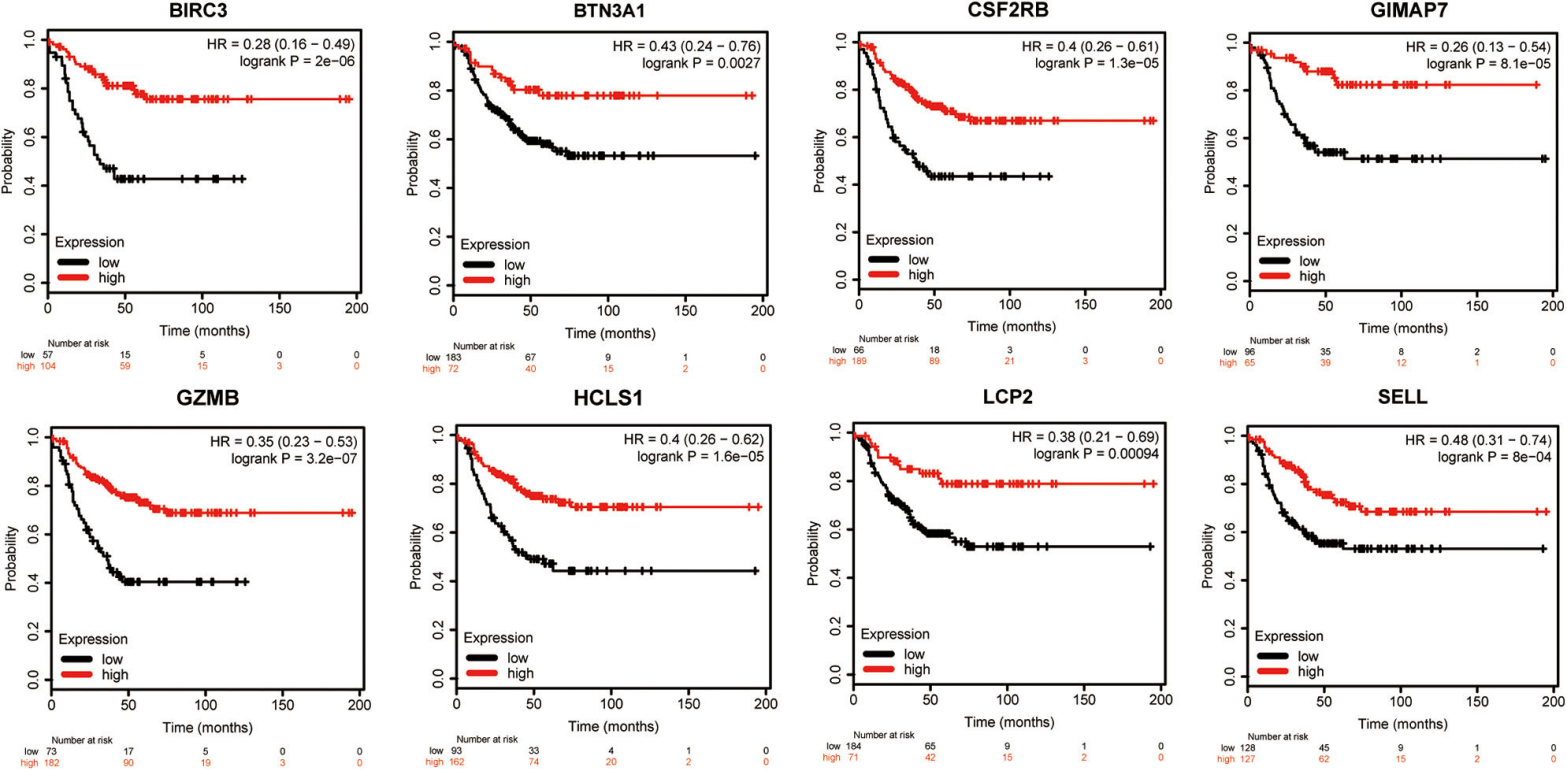
Kaplan–Meier survival curves comparing the high and low expressions of eight immune-related hub-genes.

To evaluate the value of eight immune-related genes in the progression of total breast cancer and TNBC, we assessed the correlation of eight immune-related genes with clinical survival outcomes using the Kaplan–Meier plotter tool. First, the correlation between eight immune-related gene expressions and the overall survival of breast cancer patients was analyzed (Supplementary Figure 6). Our results showed that high expression of eight immune-related genes was favorable to the overall survival of breast cancer patients. Next, the prognosis value of eight immune-related genes in patients with TNBC was also analyzed. Similarly, TNBC patients with higher expression levels of immune-related genes were significantly favorable relapse-free survival compared to those with lower expression levels of immune-related genes (Figure 7). Of interest, a combination of eight immune-related genes also displayed a remarkably favorable relapse-free survival compared to the low expression group (Supplementary Figure 7).

### Genetic Alterations of Eight Immune-Related Genes in Breast Cancer Patients

**Figure 8:**
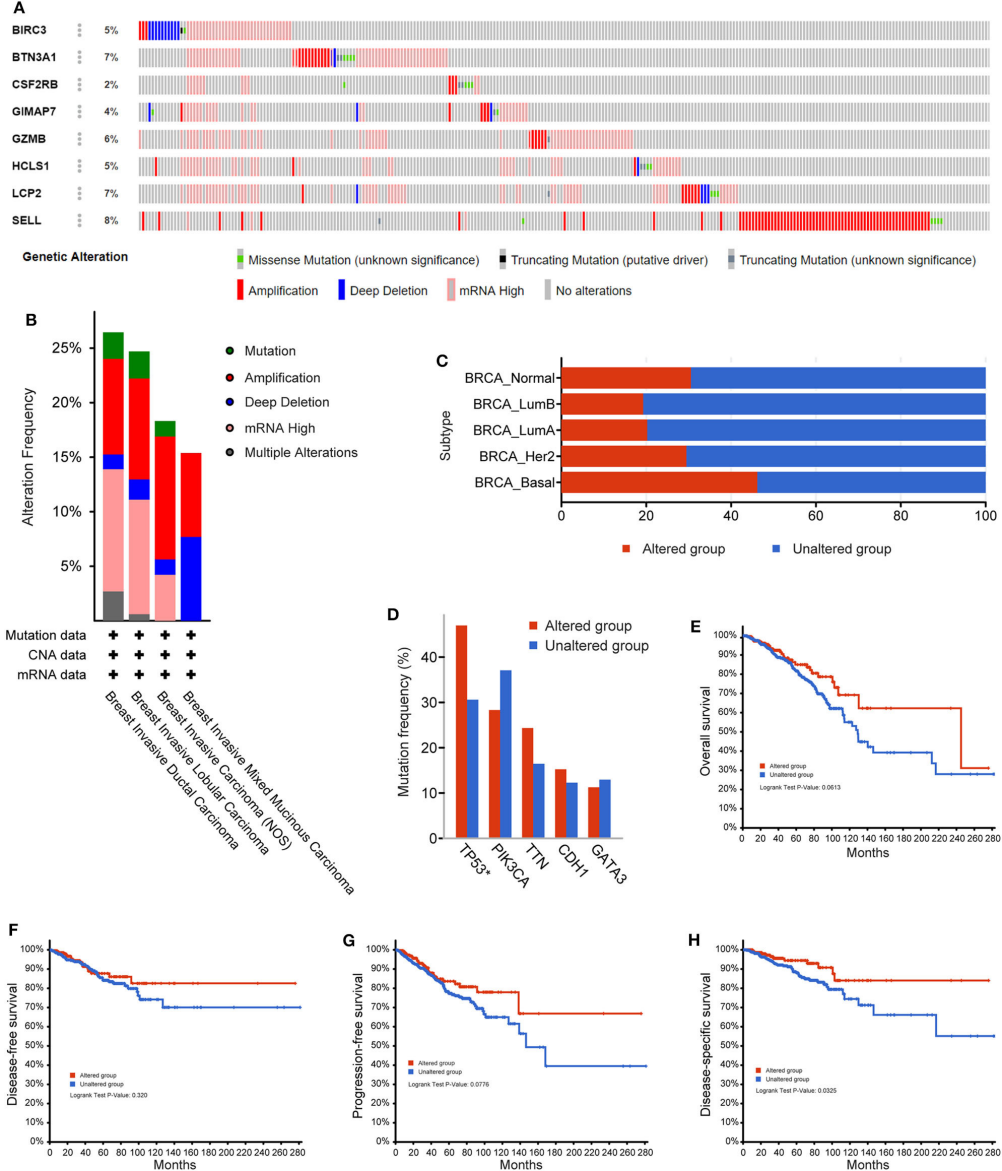
The genetic alterations of eight immune-related hub-genes in breast cancer patients. **(A)** OncoPrint summary of alterations on a query of eight immune-related hub-genes. Six types of genetic alterations were defined: Missense Mutation, Truncating Mutation (putative driver), Truncating Mutation (unknown significance), Amplification, Deep Deletion, and mRNA High. **(B)** Summary of the alteration frequency derived from mutations, copy-number alterations, and mRNA expression data in 4 types of breast invasive carcinomas in the TCGA cohort. **(C)** Summary of alterations of eight immune-related hub-genes in TCGA breast cancer subtypes. **(D)** Analysis of gene mutation co-occurrence comparing the altered group and unaltered group of eight immune-related hub-genes. Kaplan–Meier plotter shows the overall survival **(E)**, disease-free survival **(F)**, progression-free survival **(G)**, and disease-specific survival **(H)** in the altered and unaltered groups of eight immune-related hub-genes.

Next, the cBioPortal tool was used for the analysis of genetic alterations of eight immune-related hub-genes from the TCGA PanCancer Atlas dataset. Among 1,084 total samples, 994 samples were complete. The eight immune-related genes were altered in 252 patients with breast cancer. As a result, 5% BIRC3, 7% BTN3A1, 2% CSF2RB, 4% GIMAP7, 6% GZMB, 5% HCLS1, 7% LCP2, and 8% SELL were altered in six types of genetic alterations, including missense mutation, truncating mutation (putative driver), truncating mutation (unknown significance), amplification, deep deletion, and mRNA high, in the queried TCGA breast cancer samples (Figure 8A). The alteration frequency derived from mutations, copy-number alterations, and mRNA expression data was shown in 4 types of breast invasive carcinomas (Figure 8B). Among the various alterations, the amplification and high mRNA expression accounted for the two most changes in breast invasive ductal or lobular carcinomas. By comparison of subtypes of breast cancer, the basal-like subtype of breast cancer (79/172, 46.2%) accounted for the most genetic alterations than other subtypes. Most notably, genetic alterations were found to be higher in normal breast tissues (11/36, 30.56%) than LumA (101/499, 20.24%), LumB (38/197, 19.29%), and HER2 (23/76, 29.49%) subtypes of breast cancer (Figure 8C). Intriguingly, the mutation frequency analysis of the top five genes showed that a higher proportion of *TP53* mutation co-occurrence was found in the immune-related gene-altered group compared to the unaltered group (*P* = 2.271e−6; Figure 8D). Furthermore, a Kaplan–Meier plotter and log-rank test showed no significant effect of genetic alterations of eight immune-related genes on overall survival (*P* = 0.0613; Figure 8E), disease-free survival (*P* = 0.320; Figure 8F), and progression-free survival (*P* = 0.0776; Figure 8G), but significant effect on disease-specific survival (*P* = 0.0325; Figure 8F), between the genetic alteration group and the unaltered group in TCGA-breast cancer cohorts.

### Correlation of Eight Immune-Related Genes With Tumor Purity and Immune Cell Infiltration in Patients With TNBC

**Figure 9:**
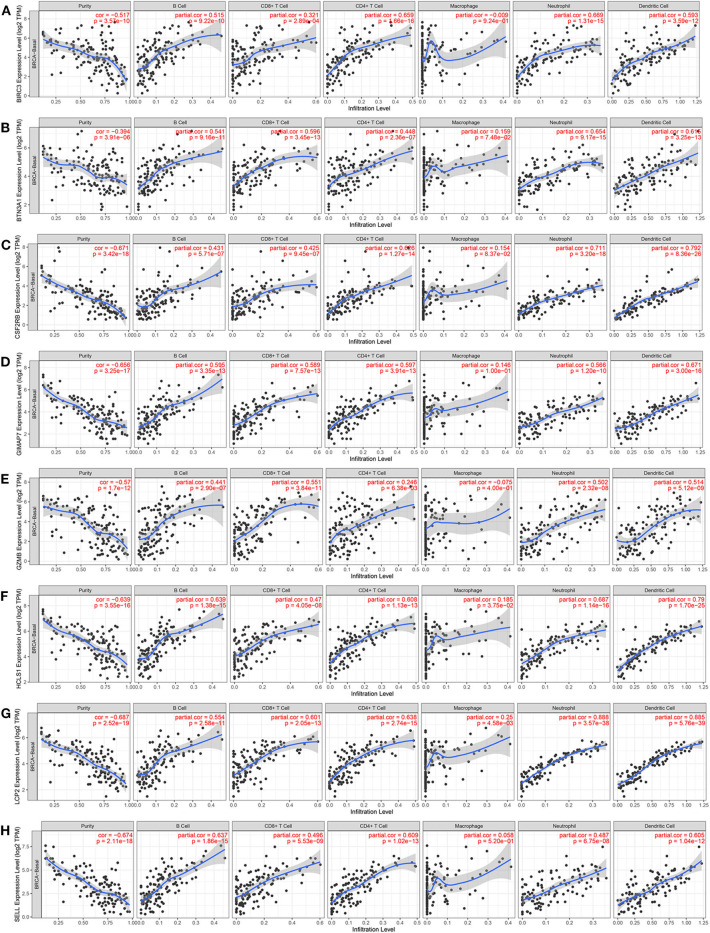
The correlation between the expression of eight immune-related hub-genes and immune cell infiltration (TIMER). The correlation between the abundance of immune cells and the expression of BIRC3 **(A)**, BTN3A1 **(B)**, CSF2RB **(C)**, GIMAP7 **(D)**, GZMB **(E)**, HCLS1 **(F)**, LCP2 **(G)**, and SELL **(H)** in TCGA-basal.

Since the functional annotation analysis revealed that the eight immune-related genes participated in the process of the immune response, next, the correlation between the expression of eight immune-related genes and immune cell infiltration in the TIMER database was further analyzed. Interestingly, high expression levels of eight immune-related genes were found to be associated with high immune cell infiltration in TNBC. A positive correlation between BIRC3 expression and the infiltration of B cells (Cor = 0.515, *p* = 9.22e−10), CD8+ T cells (Cor = 0.321, *p* = 2.89e−4), CD4+ T cells (Cor = 0.659, *p* = 1.66e−16), neutrophil (Cor = 0.669, *p* = 1.31e−15), and DCs (Cor = 0.593, *p* = 3.59e−12) were observed, while BIRC3 expression was negatively associated with the purity (Cor = −0.517, *p* = 3.57e−10; Figure 9A). Similarly, the expression of BTN3A1, CSF2RB, GIMAP7, GZMB, HCLS1, LCP2, and SELL was positively correlated with the infiltration of B cells, CD8^+^ T cells, CD4^+^ T cells, neutrophil, and DCs, but was negatively correlated with the tumor purity (Figures 9B–H). However, we did not find significant correlations of the expression of BIRC3, BTN3A1, CSF2RB, GIMAP7, GZMB, and SELL with infiltrating levels of macrophages, except for HCLS1 and LCP2.

To further investigate the relationship between eight immune-related genes and the diverse immune-infiltrating cells in TNBC, we analyzed the immune markers of immune cells with the TIMER database. Several immune markers of tumor-associated macrophages, DCs, neutrophils, T helper type 1 cells (Th1) cells, T helper type 2 cells (Th2), and regulatory T cells (Treg) were found in the basal subtype of breast cancer. The expression level of eight immune-related genes was significantly correlated with most immune markers in various immune cells (Table 2). CCL2, IL-10, CD163, VSIG4, CSF1R, FCGR2A, and FCER2 of TAMs, ITGAX, CD1C, NRP1, and THBD of DCs, CCR7, ITGAM, and CD59 of neutrophils, STAT4, TBX21, and CD4 of Th1 cells, CCR4 and CCR4 of Th2 cells, and FOXP3, STAT5B, and TGFB1 of Treg cells were positively correlated with eight immune-related genes. These results further indicate that eight immune-related genes may enhance immune activities in the TNBC microenvironment.

**Table 2:** Correlation between eight immune-related hub-genes and the biomarkers of immune cells.

**Immune cells**	**Gene marker**	**BIRC3**		**BTN3A1**		**CSF2RB**		**GIMAP7**		**GZMB**		**HCLS1**		**LCP2**		**SELL**	
		**Cor**	***P***	**Cor**	***P***	**Cor**	***P***	**Cor**	***P***	**Cor**	***P***	**Cor**	***P***	**Cor**	***P***	**Cor**	***P***
TAM	CCL2	0.553	^***^	0.529	^***^	0.703	^***^	0.643	^***^	0.651	^***^	0.643	^***^	0.773	^***^	0.667	^***^
	IL10	0.637	^***^	0.634	^***^	0.757	^***^	0.671	^***^	0.616	^***^	0.705	^***^	0.812	^***^	0.666	^***^
	CD163	0.626	^***^	0.629	^***^	0.777	^***^	0.656	^***^	0.605	^***^	0.726	^***^	0.827	^***^	0.596	^***^
	VSIG4	0.546	^***^	0.448	^***^	0.703	^***^	0.563	^***^	0.527	^***^	0.657	^***^	0.744	^***^	0.486	^***^
	CSF1R	0.684	^***^	0.644	^***^	0.852	^***^	0.730	^***^	0.586	^***^	0.835	^***^	0.896	^***^	0.675	^***^
	FCGR2A	0.477	^***^	0.596	^***^	0.670	^***^	0.593	^***^	0.572	^***^	0.681	^***^	0.775	^***^	0.503	^***^
	FCER2	0.395	^***^	0.321	^***^	0.555	^***^	0.681	^***^	0.479	^***^	0.621	^***^	0.565	^***^	0.770	^***^
DCs	ITGAX	0.648	^***^	0.598	^***^	0.852	^***^	0.741	^***^	0.619	^***^	0.825	^***^	0.890	^***^	0.714	^***^
	CD1C	0.497	^***^	0.378	^***^	0.623	^***^	0.604	^***^	0.388	^***^	0.621	^***^	0.605	^***^	0.667	^***^
	NRP1	0.184	^*^	0.309	^***^	0.338	^***^	0.356	^***^	0.130	0.120	0.337	^***^	0.420	^***^	0.267	^**^
	THBD	0.208	^*^	0.208	^*^	0.435	^***^	0.491	^***^	0.210	^*^	0.394	^***^	0.437	^***^	0.478	^***^
Neutrophils	CCR7	0.656	^***^	0.517	^***^	0.777	^***^	0.870	^***^	0.702	^***^	0.781	^***^	0.813	^***^	0.950	^***^
	ITGAM	0.720	^***^	0.568	^***^	0.783	^***^	0.634	^***^	0.664	^***^	0.714	^***^	0.815	^***^	0.565	^***^
	CD59	0.335	^***^	0.318	^***^	0.174	^*^	0.171	^*^	0.112	0.180	0.278	^***^	0.237	^**^	0.149	0.070
Th1	STAT4	0.743	^***^	0.689	^***^	0.771	^***^	0.878	^***^	0.748	^***^	0.775	^***^	0.868	^***^	0.841	^***^
	TBX21	0.657	^***^	0.704	^***^	0.780	^***^	0.864	^***^	0.866	^***^	0.811	^***^	0.885	^***^	0.819	^***^
	CD4	0.738	^***^	0.678	^***^	0.899	^***^	0.844	^***^	0.702	^***^	0.903	^***^	0.960	^***^	0.807	^***^
Th2	GATA3	0.142	0.090	0.006	0.940	0.075	0.37	0.112	0.180	0.104	0.2	0.090	0.290	0.122	0.150	0.171	^*^
	CXCR4	0.231	^**^	0.116	0.170	0.116	0.17	0.115	0.170	−0.006	0.940	0.168	^*^	0.132	0.120	0.047	0.580
	CCR4	0.661	^***^	0.591	^***^	0.787	^***^	0.806	^***^	0.556	^***^	0.760	^***^	0.835	^***^	0.847	^***^
	CCR8	0.867	^***^	0.644	^***^	0.777	^***^	0.777	^***^	0.593	^***^	0.753	^***^	0.853	^***^	0.817	^***^
Treg	FOXP3	0.653	^***^	0.614	^***^	0.784	^***^	0.798	^***^	0.685	^***^	0.791	^***^	0.845	^***^	0.850	^***^
	STAT5B	0.208	^*^	0.292	^***^	0.283	^***^	0.356	^***^	0.063	0.450	0.337	^***^	0.283	^***^	0.323	^***^
	TGFB1	0.429	^***^	0.487	^***^	0.681	^***^	0.619	^***^	0.352	^***^	0.664	^***^	0.661	^***^	0.547	^***^

TAM, tumor-associated macrophages; DCs, dendritic cells; Th1/2, T helper type 1/2 cells; Treg, regulatory T cells; Cor, correlation coefficient; P, P-value;

* P < 0.05;

** P < 0.01;

*** *P < 0.001*.

### Eight Immune-Related Genes Were Positively Correlated With the Expression of PD-L1, PD-1, and CTLA4 in TCGA-BRCA Samples

**Figure 10:**
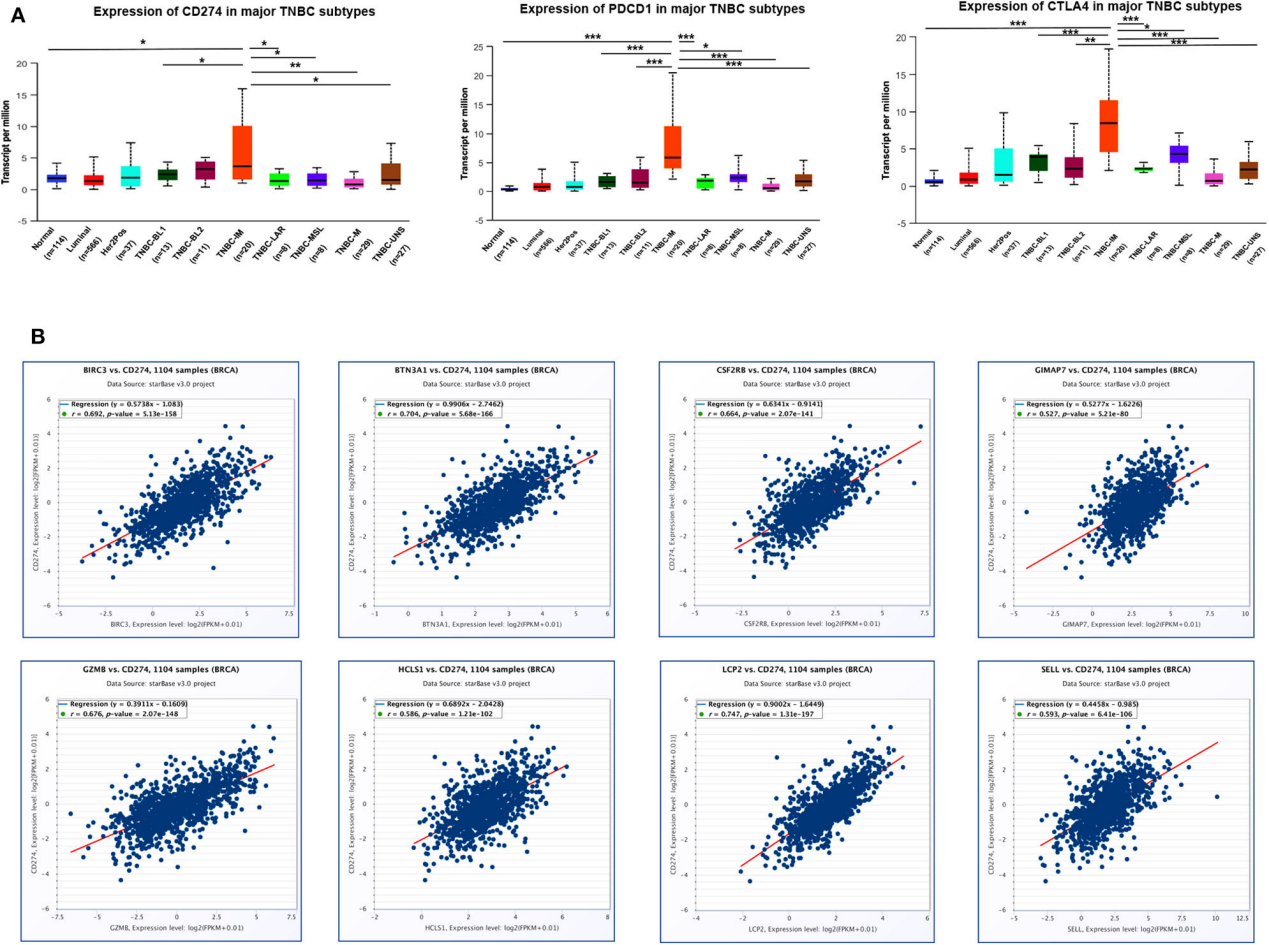
The correlation of eight immune-related hub-genes with the expression of PD-L1, PD-1, and CTLA4 in TCGA-BRCA samples. **(A)** The transcription level of PD-L1 (CD274), PD-1(PDCD1), and CTLA4 in TNBC subtypes (UALCAN). *p < 0.05, **p < 0.01, ***p < 0.001. **(B)** The correlation between the expression of eight immune-related hub-genes and PD-L1 (CD274) in the TCGA-BRCA cohort (starBase).

Since immune checkpoint blockers may constitute an effective therapeutic strategy, next, we investigated the immune checkpoint-related molecules such as PD-L1 (CD274), PD-1 (PDCD1), and CTLA4 (cytotoxic T-lymphocyte associated protein 4) in TNBC subtypes. Data from TCGA showed that the highest level of PD-L1, PD-1, and CTLA4 gene expression was found in the IM subtype of TNBC compared with other subtypes and normal breast tissues (Figure 10A), indicating that patients with high IM subtype may benefit from the therapy of checkpoint blockers. Since eight immune-related genes were also elevated in TNBC IM patients, we next examined the correlation of eight immune-related genes with checkpoint-related genes using TCGA breast cancer datasets. Intriguingly, a positive correlation of mRNA expression was observed between eight immune-related genes and PD-L1 (Figure 10B), PD-1 (Supplementary Figure 8), and CTLA4 (Supplementary Figure 9) in patients with breast cancer.

## Discussion

The current study used WGCNA to analyze mRNA microarray datasets with 107 TNBC patients and identified a module of TNBC highly related to survival and metastasis. Functional annotations containing GO terms and KEGG pathways were displayed in this interesting module and revealed 558 genes within the module participated in the process of the immune response. Furthermore, among 50 hub-genes in the module, eight hub-genes were immune-related and associated with survival in the discovery and validation datasets. Finally, comprehensive analyses of eight immune-related hub-genes at the levels of mRNA and protein expression, genetic alterations, and tumor immune microenvironment with validation from qRT-PCR and immunohistochemistry and data mining from Oncomine, UALCAN, TCGA, starBase, Kaplan-Meier plotter, cBioPortal, TIMER databases were conducted.

WGCNA of network modeling is a novel approach that relies on complex statistical algorithms and can determine complex biological relationships between the biological networks and their phenotypes (23). This powerful method has been applied to study many refractory diseases, such as Alzheimer's disease (41), familial combined hyperlipidemia (42), and breast cancer (43). The general advancements in genome sequencing and other “omics” technologies have promoted an understanding of the biological heterogeneity of TNBC and have revealed more intrinsic molecular TNBC subtypes (44). When we make a treatment plan such as primary surgery, chemotherapy, radiotherapy, endocrine therapy, and biotherapy, the specific molecular subtype should be taken into account as described in the literature (45). However, nearly 30–40% of patients with breast cancer at the early-stage may ultimately develop metastatic lesions, recurrence, or resistance to chemotherapy (46). Furthermore, both TNBC and basal-like breast cancers have a higher rate of local relapse and are more likely to metastasize to viscera rather than bone, particularly to the brain and lungs (47). To date, there are no practicable biomarkers to specifically distinguish the subtypes of TNBC. The current study unveiled eight immune-related hub-genes in TNBC patients and these potential biomarkers may be the key to manage early TNBC treatment. It has been shown that blockade therapies can be applied to patients who have elevated biomarkers (48).

Among 11 co-expression modules constructed in this study, the blue module containing 558 genes was most correlated with the survival status and metastasis of TNBC and was most enriched in the immune process and defense response defined by the functional enrichment analysis of GO terms. Furthermore, the KEGG pathway analysis showed that the most significant items were antigen processing and presentation. Therefore, we hypothesized that the blue module affected TNBC survival and metastasis mainly through the dysfunction of the immune system. Indeed, the mRNA expression of all eight hub-genes was elevated in the immunomodulatory subtype of TNBC compared with other subtypes of TNBC. Our immunohistochemistry study validated the protein expression of eight immune-related hun-genes in tissue samples derived from patients with TNBC and non-TN breast cancer. The high expression levels of eight proteins were found in TNBC tissues compared to non-TN breast cancer tissues, indicating that these eight proteins may play important roles in patients with TNBC.

In recent years, immunotherapy has been a promising therapy for cancer (49). The PD-L1 antibody such as Atezolizumab presented outstanding outcomes in the treatment of TNBC patients who had PD-L1 positive expression (50). In the immune equilibrium phase, it is capable to reduce the risk of tumor metastasis or to maintain tumor dormancy although the immune system is unable to eliminate the tumor (51). Furthermore, the percentages of tumor-infiltrating lymphocytes (TILs), CD8^+^ cells, and CD4^+^ T cells are lower in the metastatic tumors of TNBC compared to primary tumors (52), implying that immune escape may play an important role in tumor metastasis. Previous studies have indicated that the immune response of TNBC patients makes a positive impact on therapy response with improved progression-free survival (53, 54).

The current study demonstrated that these eight immune-related hub-genes were upregulated in immune cells and acted as favorable factors of the survival of patients with breast cancer or TNBC. Accumulated studies have indicated that a high ratio of TILs could act as an independent factor to improved patient survival in breast cancer (55–57). CD8^+^ T cell-mediated cytotoxic effect can promote the growth of endogenous CD8^+^ and CD4^+^ T cells and immunity-associated cells, thus facilitating their antitumor function in the tumor microenvironment (58). Genetic alterations were more common in TNBC, which was in agreement with our findings in TCGA TNBC patients. High levels of genomic mutations of *TP53* (82%) and *PIK3CA* (10%), the two most frequently mutated somatic genes, occur in TNBC (59). Interestingly, our results also showed that the alterations of eight immune-related genes were co-occurrence with *TP53* mutation, indicating a potential regulatory mechanism.

The immune microenvironment of cancer cells plays an important role in inhibiting tumor proliferation or promoting tumor progression (60). It has been shown that the IM subtype of TNBC is characterized by high levels of immune antigens and genes involved in cytokine and core immune signal transduction pathways (59). A more recent report shows that the IM gene signature is an indicator of the presence of TILs and redefines the IM subtype as a modifier of the other subtypes rather than a single subtype (61). The most noticeable difference between IM subtype and other TNBC subtype seems to lie in high expression levels of immune-related genes. Our eight immune-related hub-genes screened by WGCNA were more enriched in the TNBC IM subtype and positively correlated with the infiltration of the TILs and other immune cells. These data indicate that eight immune-related hub-genes were not only as prognostic indicators but also reflect “immune-hot” status in the TNBC IM subtype. It has been reported that mRNA expression levels of immune checkpoint inhibitor genes such as PD-L1, PD-1, CTLA4, and IDO1 are higher in the IM subtype (13). Similarly, we also found a higher level of PD-L1, PD-1, and CTLA4 transcription in the IM subtype of TNBC through the analysis of TCGA datasets. Of noted, a surprising result of a phase III trial indicated that anti-PD-L1 (Atezolizumab) combined with nanoparticle albumin-bound (nab)-paclitaxel prolonged progression-free survival in metastatic TNBC with PD-L1-positive subgroup (50). Furthermore, our eight immune-related hub-genes had a positive relationship with PD-L1, PD-1, and CTLA4, implying that patients with these immune-related signatures may benefit from immune checkpoint inhibitors.

The current study using TCGA datasets analyzed the association of the expression of eight immune-related hub-genes with the clinical features, such as age, stage, menopausal status, and lymph node metastasis. Among these eight hub-genes, BIRC3 expression was found to be higher in younger, pre-menopause patients with nodal metastatic BC at early-stage. The expression of GZMB, HCLS1, HCP2, and SELL was higher in metastatic patients. SELL expression was also found to be higher in younger patients with breast cancer. However, the expression of BTN3A1, CSF2RB, and GIMAP7 was not associated with clinical features in patients with breast cancer, suggesting the irrelevance of these immune-related genes to clinical features.

BTN3A1 is the main isoform of the butyrophilin 3A (BTN3A, CD277) family and can directly bind phosphor-antigens (62), activating the Vγ9Vδ2 T cells in colorectal cancer microenvironment for the anti-tumor response of zoledronate (63). BIRC3, an inhibitor of apoptosis protein by inhibiting caspases cascade, serves as a putative biomarker for patients with oesophageal adenocarcinoma (64) and is associated with therapeutic resistance in glioblastoma and breast cancer cells (65, 66). CSF2RB is the common beta chain receptor for the GM-CSF, IL-3, and IL-5 activation (67) and a rare genetic variant of CSF2RB (rs16997517) is related to a decreased risk of squamous cell cervical cancer (68). GZMB exists as cytoplasmic granules in cytotoxic T cells and NK cells that directly kills the target cells in a perforin-dependent manner (69). GZMB contributes to the invasive and metastatic phenotypes in colorectal cancer and urothelial carcinoma (70, 71). A lower level of GZMB is associated with shorter relapse-free survival of TNBC patients, whereas a higher level of GZMB is associated with improved cancer-specific survival of colorectal cancer patients (72, 73). HCLS1 is expressed mainly in hematopoietic cells (74), which regulates the migration of leukemic cells and may be a promising target for the treatment of chronic lymphocytic leukemia (75, 76). LCP2 acts as a substrate to activate the T cell antigen receptor signaling pathway and is expressed in chronic lymphocytic leukemia cells (77). SELL is expressed in inflammatory cells including T cells and B cells and serves as a biomarker to predict the metastatic risk of high-grade urothelial carcinoma (78). GIMAP7 belongs to the GTP-binding superfamily and the prognostic role of GIMAP7 in cancer has not been explored yet. Nevertheless, all eight immune-related hub-genes seem to be important because of the involvement of the immune response in cancer patients.

To our knowledge, this is the first study to identify several gene signatures that were closely related to the IB subtype of TNBC. However, some limitations still exist. First, the detailed clinical information of metastasis status was unavailable in the validation dataset. Second, most TNBC datasets in the public repository database lack clinically relevant information. Therefore, the robustness of eight immune-related hub-genes should be confirmed in prospective, multiple-center, large-sample cohorts in the future. Practically, it is better to have another independent study to validate our results.

In summary, eight immune-related genes as molecular signatures are unveiled in the IM subtype of TNBC. These immune-related genes are upregulated in the TNBC tissues and cell lines as well as the IM subtype of patients, which are associated with the survival and metastasis of TNBC, acting as favorable factors for the survival of patients with TNBC. We also reveal a positive correlation of the expression of eight immune-related genes with the infiltration of immune cells in TNBC. Moreover, eight immune-related genes have a close relationship with checkpoint inhibitor genes such as PD-L1, PD-1, and CTLA4, suggesting that TNBC patients in the IM subtype may benefit from checkpoint inhibitor therapies. Large-scale TNBC genomics research and subsequently functional studies are required in the future.

## Data Availability Statement

The datasets presented in this study can be found in online repositories. The names of the repository/repositories and accession number(s) can be found in the article/Supplementary Material.

## Ethics Statement

The studies involving human participants were reviewed and approved by The Ethics Committee of Jinshan Hospital, Fudan University (JYLLKY-2017-11-01). The patients/participants provided their written informed consent to participate in this study.

## Author Contributions

JZ and GX developed the idea and designed the research. JZ, XX, and WG analyzed the data. JZ, XL, and TM performed cell culture and PCR. JZ wrote the draft of the manuscript. GX wrote and edited the manuscript. All authors contributed to the article and approved the submitted version.

## Conflict of Interest

The authors declare that the research was conducted in the absence of any commercial or financial relationships that could be construed as a potential conflict of interest.

## Supplementary Material

Supplementary Figure 1 Sample clustering analysis to detect outliers based on the dataset GSE58812.

Supplementary Figure 2 The correlation between the expression of eight immune-related hub-genes and nodal metastasis status in the TCGA-BRCA cohort. ^*^*p* < 0.05, ^**^*p* < 0.01, ^***^*p* < 0.001.

Supplementary Figure 3 The correlation between the expression of eight immune-related hub-genes and patient age in the TCGA-BRCA cohort. ^*^*p* < 0.05, ^**^*p* < 0.01, ^***^*p* < 0.001.

Supplementary Figure 4 The correlation between the expression of eight immune-related hub-genes and cancer stage in the TCGA-BRCA cohort. ^*^*p* < 0.05, ^**^*p* < 0.01, ^***^*p* < 0.001.

Supplementary Figure 5 The correlation between the expression of eight immune-related hub-genes and the menopausal status of patients in the TCGA-BRCA cohort. ^*^*p* < 0.05.

Supplementary Figure 6 Kaplan–Meier survival curves for the overall survival of patients with breast cancer correlated with the high and low expressions of eight immune-related hub-genes.

Supplementary Figure 7 Kaplan–Meier survival curves for the relapse-free survival of patients with breast cancer correlated with the high and low expressions of combined eight immune-related hub-genes.

Supplementary Figure S8 The correlation between the expression of eight immune-related hub-genes and PD-1 (PDCD1) in the TCGA-BRCA cohort (starBase).

Supplementary Figure S9 The correlation between the expression of eight immune-related hub-genes and CTLA4 in the TCGA-BRCA cohort (starBase).

Supplementary Table 1 Sequences of PCR primer. The GenBank Accession number is shown: BTN3A1 (NM_001145), BIRC3 (NM_001165), CSF2RB (NM_000395), GIMAP7 (NM_153236), GZMB (NM_004131), HCLS1 (NM_005335), LCP2 (NM_005565), SELL (NM_000655), and GAPDH (NM_001256799). nt, nucleotide.

Supplementary Table 2 Number of genes in the 11 modules.

Supplementary Table 3 Top 50 hub genes.
