# Supplementary figures and images for: Transcriptome-Based Network Analysis Unveils Eight Immune-Related Genes as Molecular Signatures in the Immunomodulatory Subtype of Triple-Negative Breast Cancer
Source: Front Oncol. 2020 Sep 18;10:1787. doi: 10.3389/fonc.2020.01787 (PMC7530237; doi:10.3389/fonc.2020.01787)

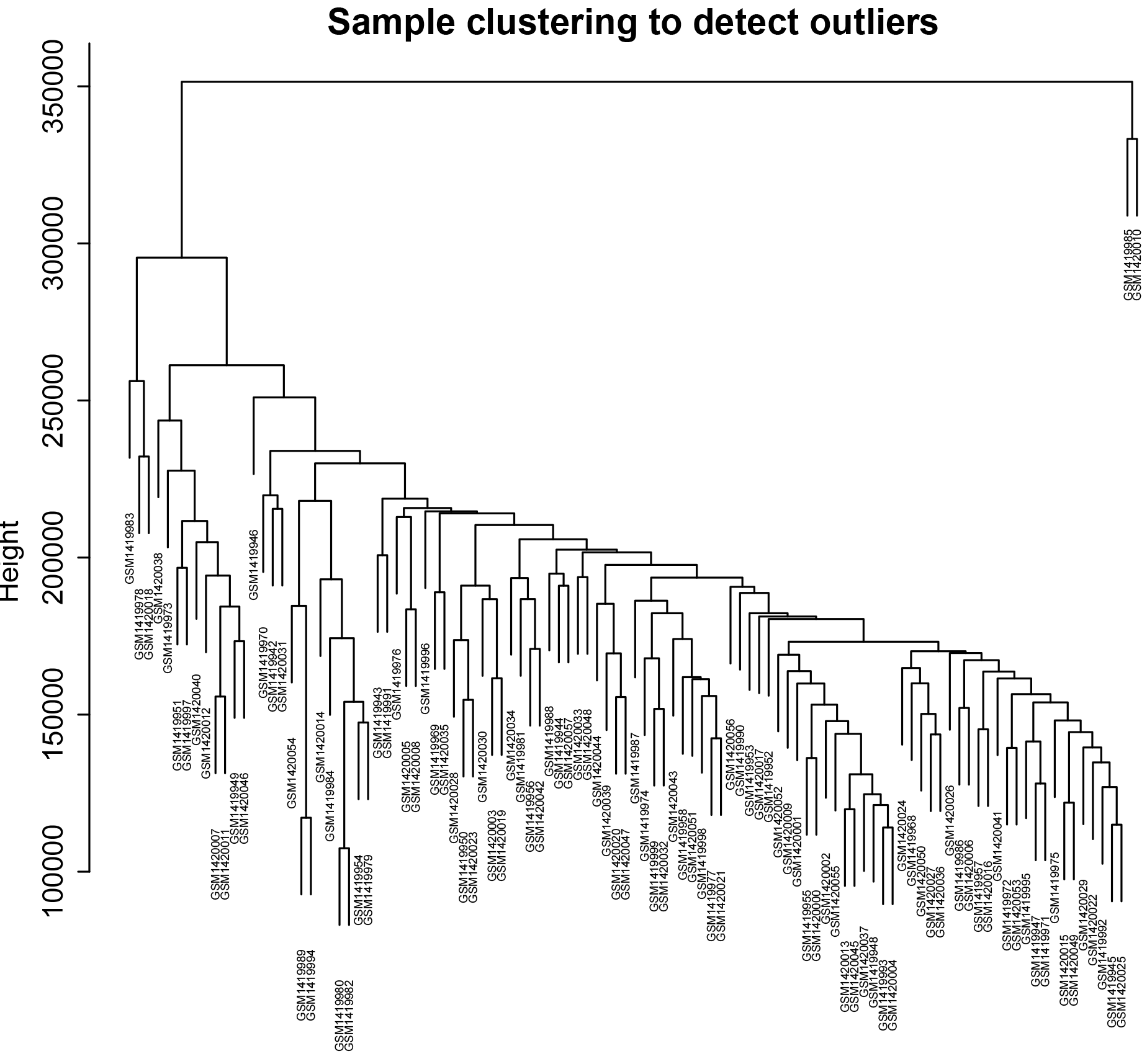

Supplement: Supplementary Figure 1 — Sample clustering analysis to detect outliers based on the dataset GSE58812. [file Image_1.TIF]

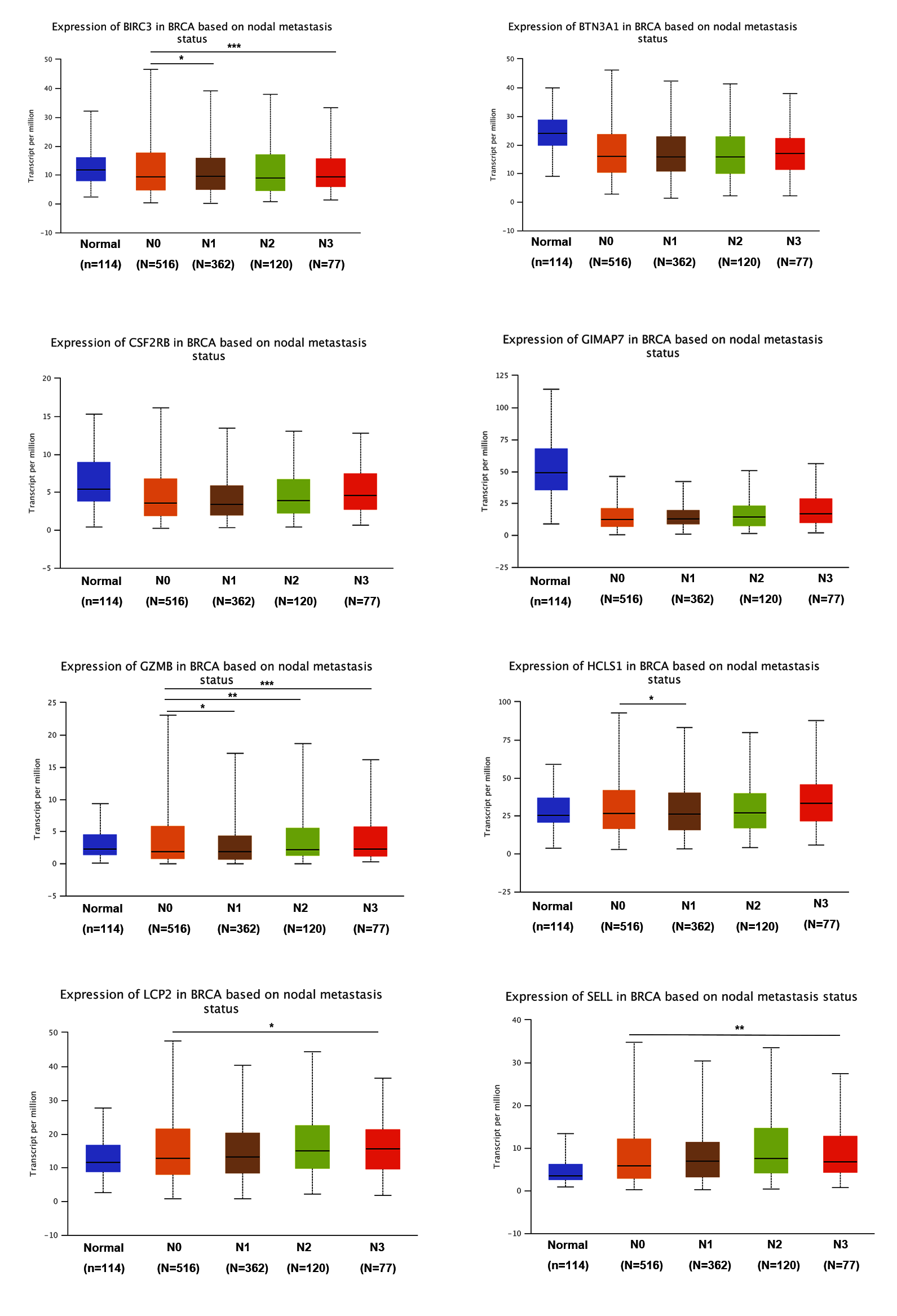

Supplement: Supplementary Figure 2 — The correlation between the expression of eight immune-related hub-genes and nodal metastasis status in the TCGA-BRCA cohort. *p < 0.05, **p < 0.01, ***p < 0.001. [file Image_2.TIF]

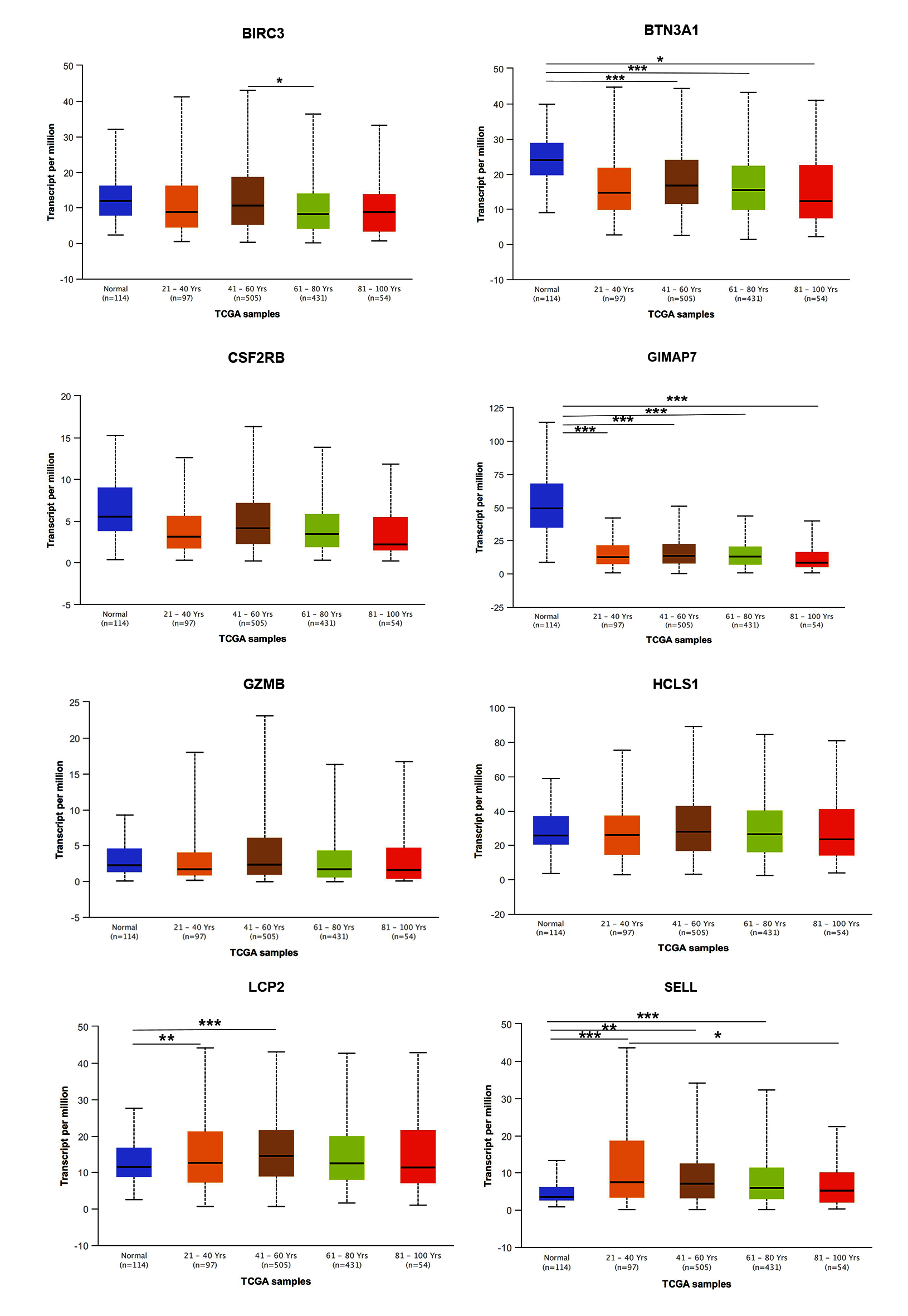

Supplement: Supplementary Figure 3 — The correlation between the expression of eight immune-related hub-genes and patient age in the TCGA-BRCA cohort. *p < 0.05, **p < 0.01, ***p < 0.001. [file Image_3.TIF]

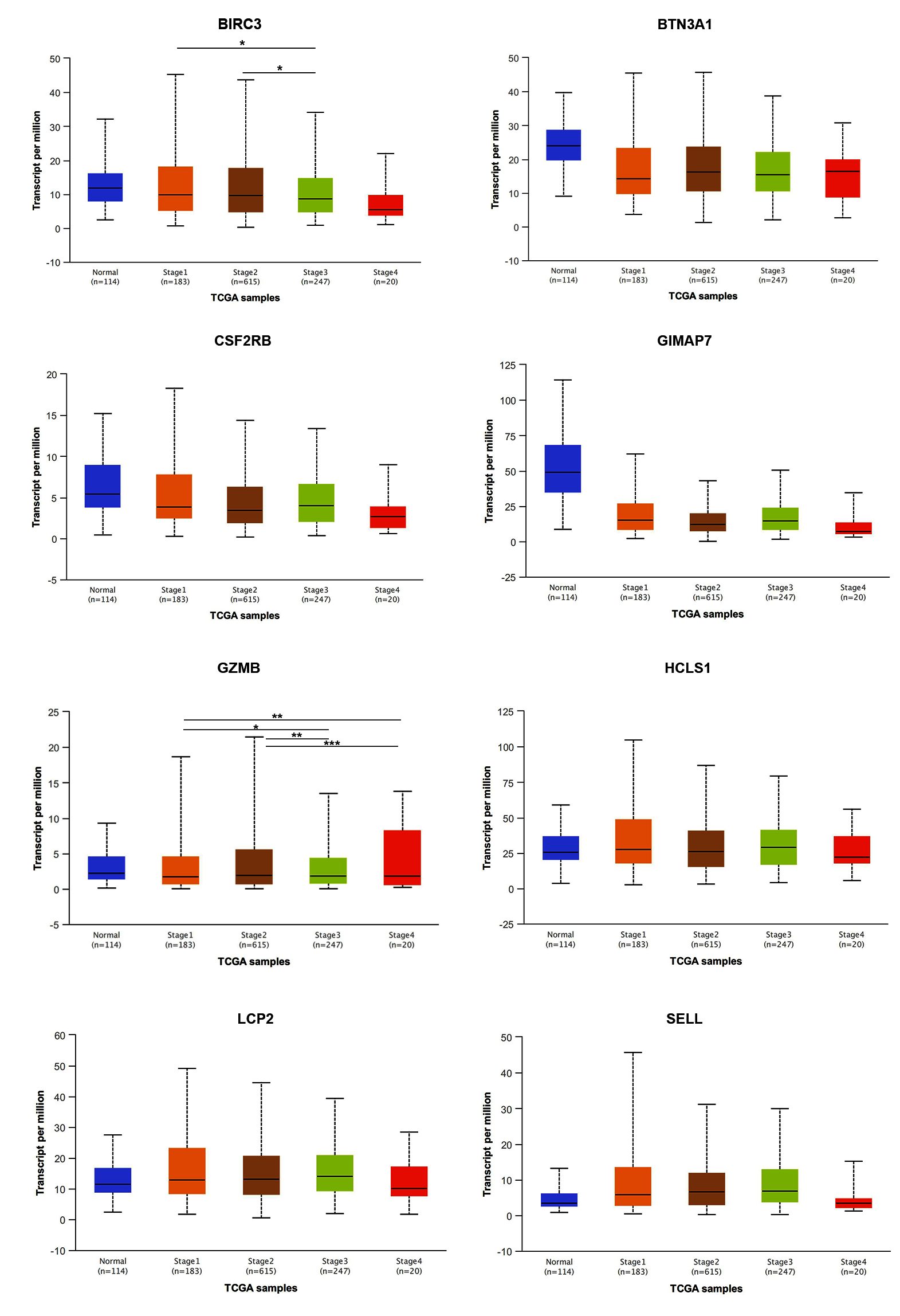

Supplement: Supplementary Figure 4 — The correlation between the expression of eight immune-related hub-genes and cancer stage in the TCGA-BRCA cohort. *p < 0.05, **p < 0.01, ***p < 0.001. [file Image_4.TIF]

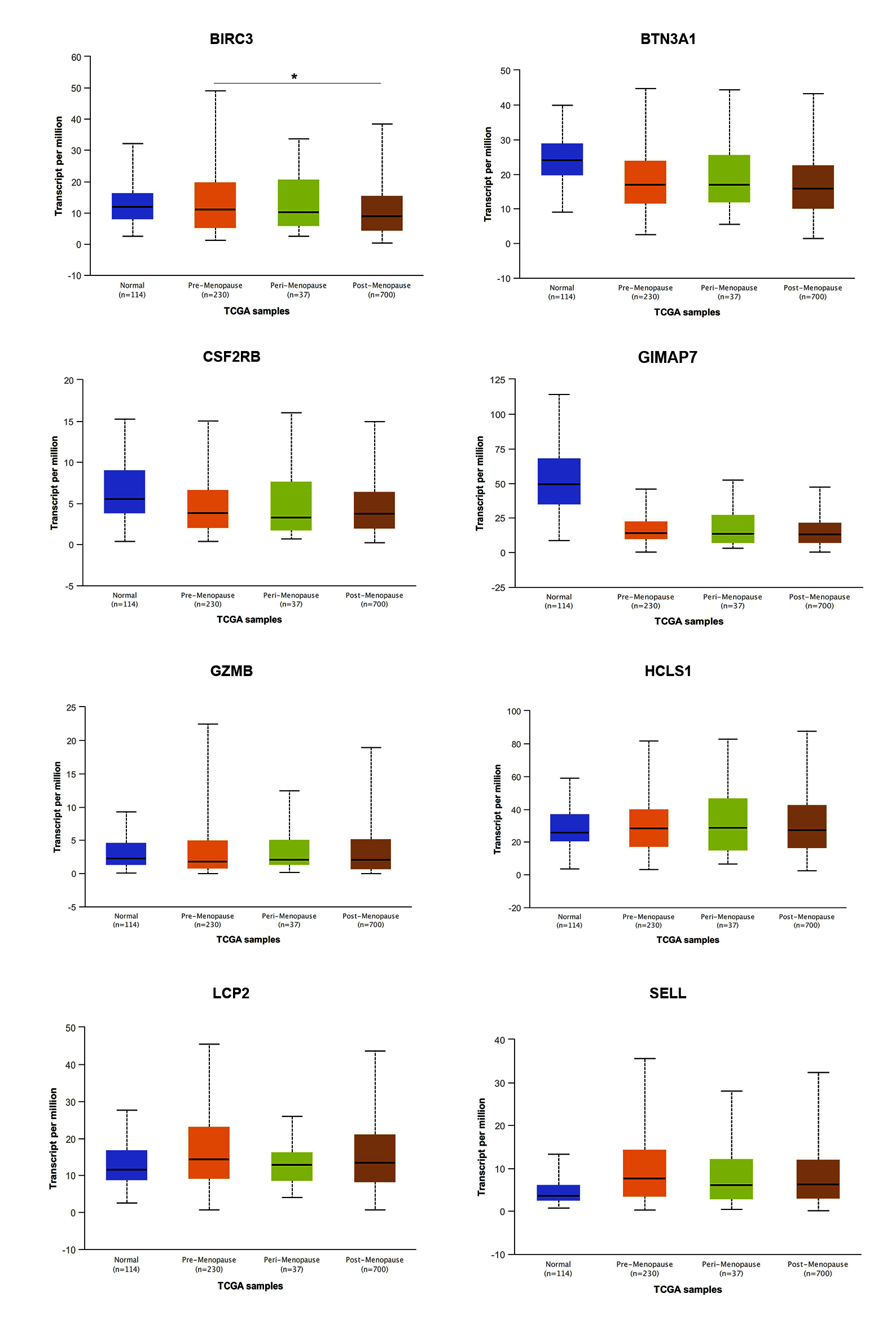

Supplement: Supplementary Figure 5 — The correlation between the expression of eight immune-related hub-genes and the menopausal status of patients in the TCGA-BRCA cohort. *p < 0.05. [file Image_5.TIF]

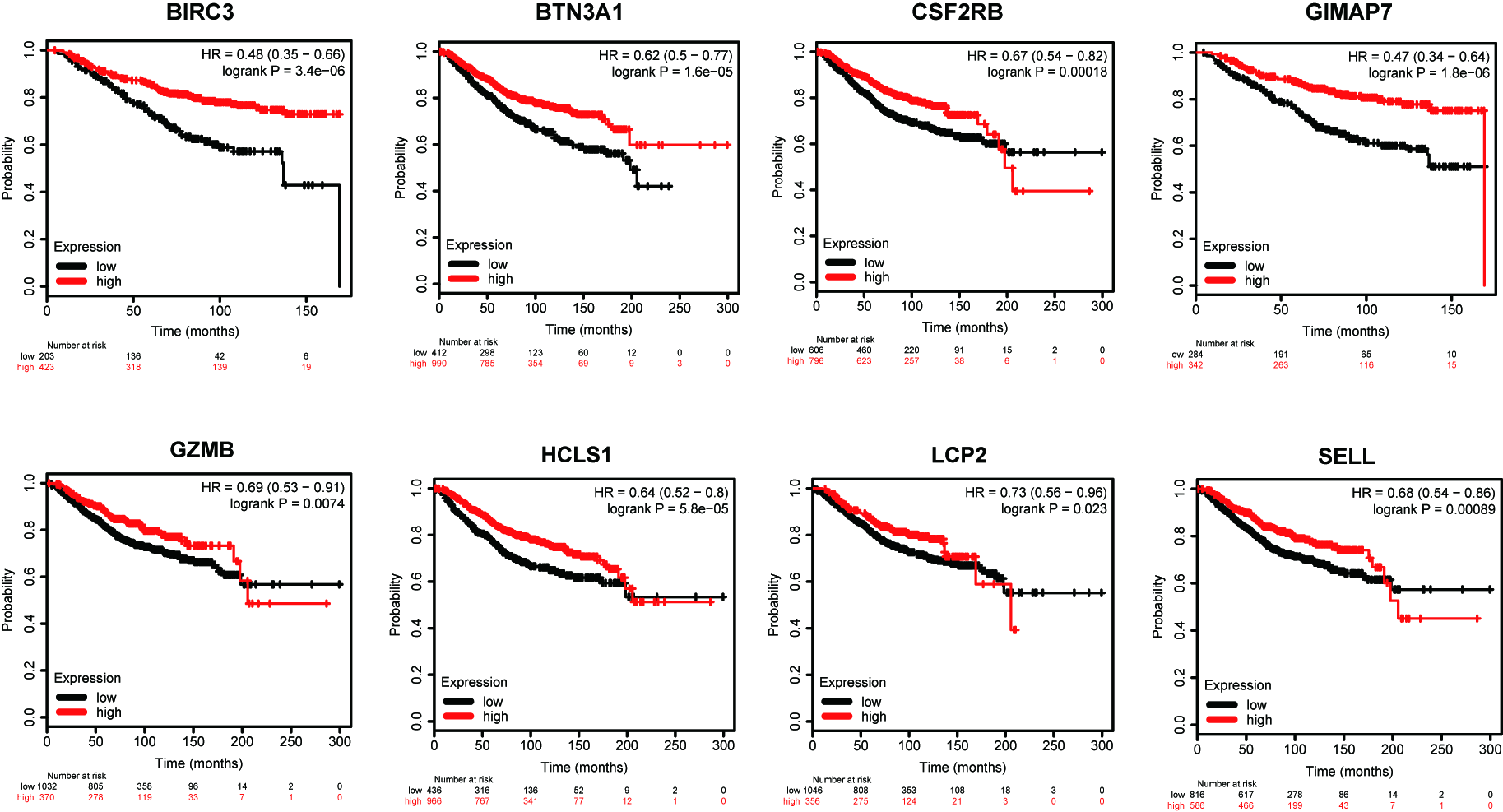

Supplement: Supplementary Figure 6 — Kaplan–Meier survival curves for the overall survival of patients with breast cancer correlated with the high and low expressions of eight immune-related hub-genes. [file Image_6.TIF]

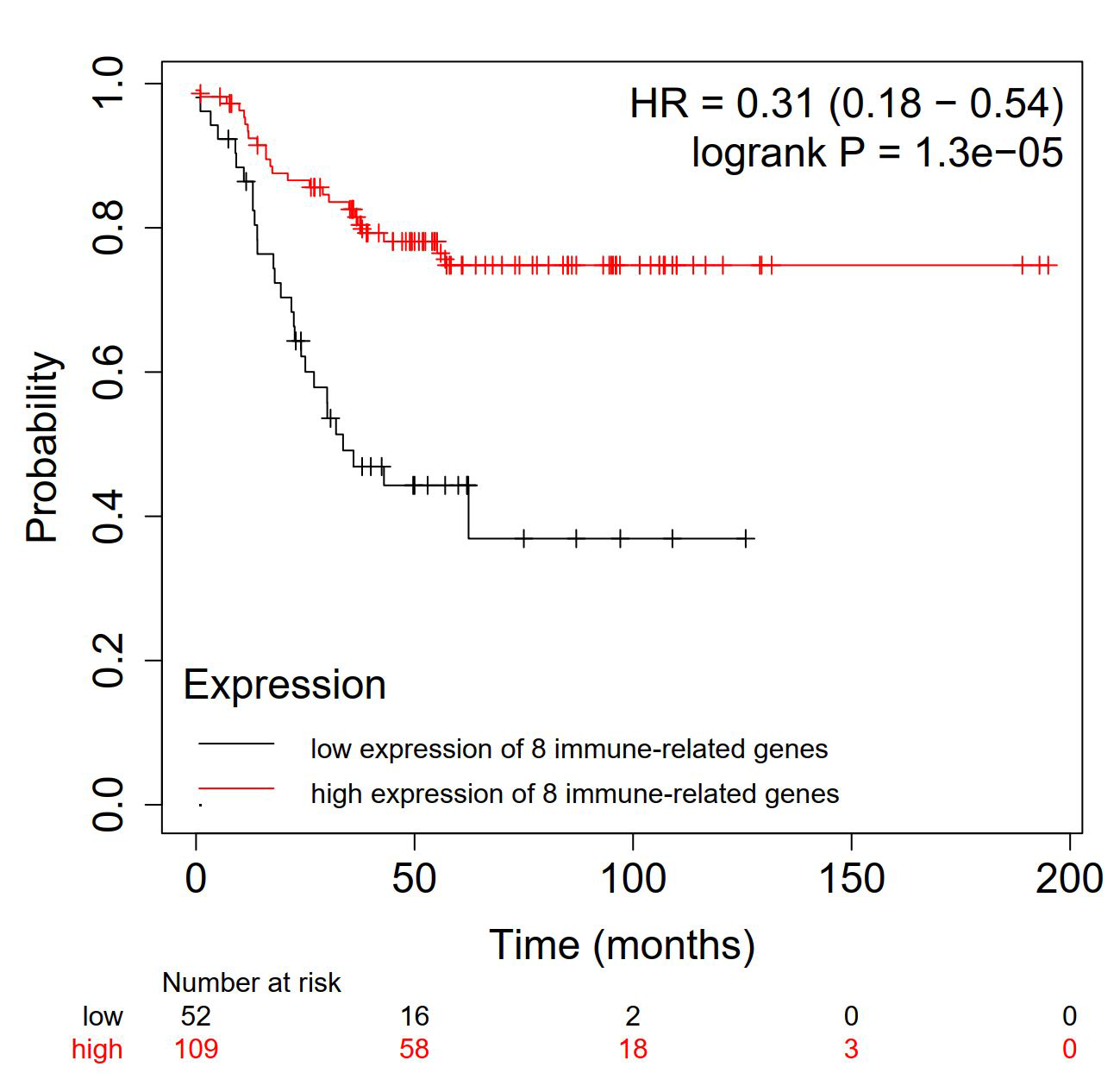

Supplement: Supplementary Figure 7 — Kaplan–Meier survival curves for the relapse-free survival of patients with breast cancer correlated with the high and low expressions of combined eight immune-related hub-genes. [file Image_7.TIF]

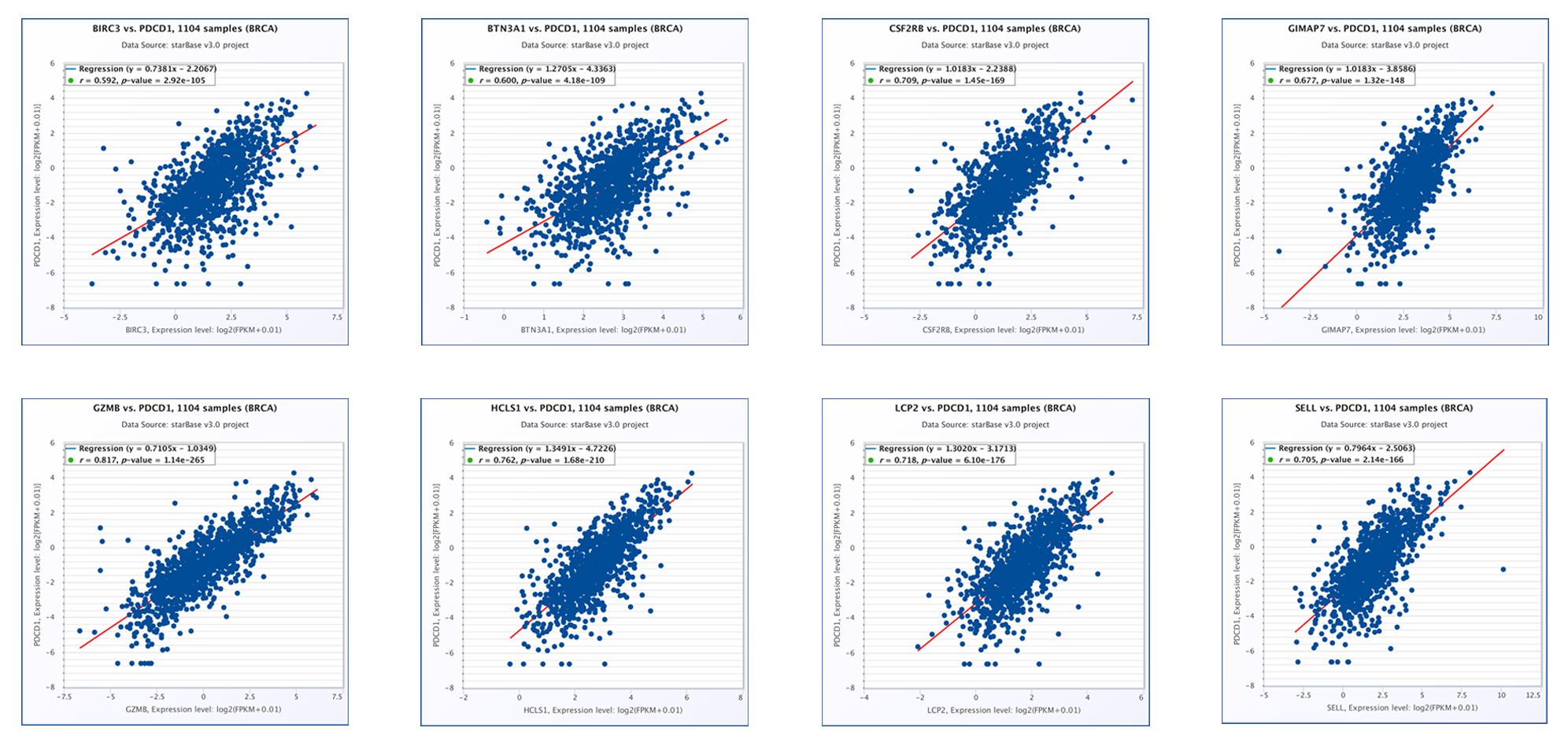

Supplement: Supplementary Figure S8 — The correlation between the expression of eight immune-related hub-genes and PD-1 (PDCD1) in the TCGA-BRCA cohort (starBase). [file Image_8.TIF]

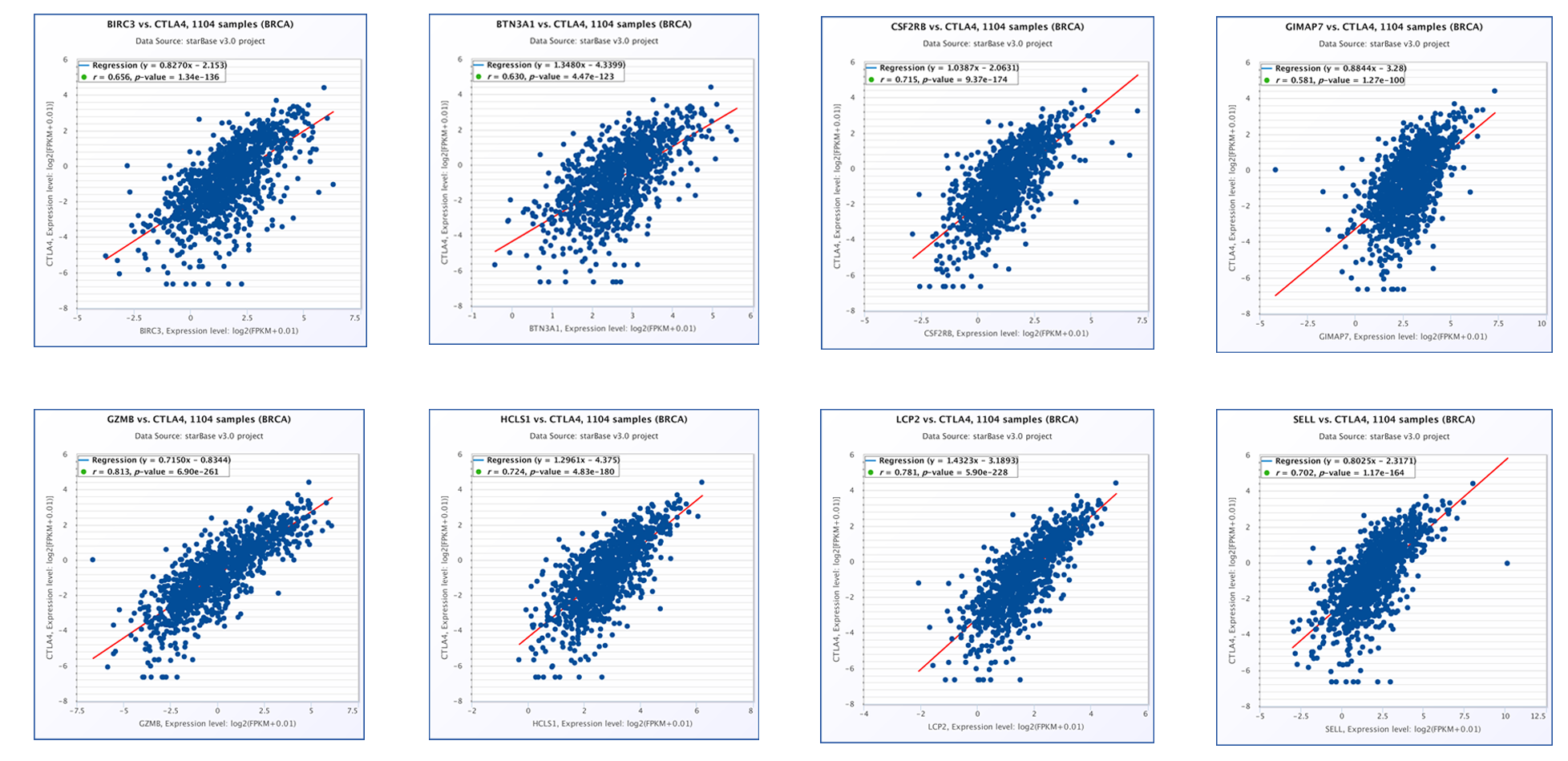

Supplement: Supplementary Figure S9 — The correlation between the expression of eight immune-related hub-genes and CTLA4 in the TCGA-BRCA cohort (starBase). [file Image_9.TIF]
