# Supplementary material for: Transcriptome-Based Network Analysis Unveils Eight Immune-Related Genes as Molecular Signatures in the Immunomodulatory Subtype of Triple-Negative Breast Cancer
Source: Front Oncol. 2020 Sep 18;10:1787. doi: 10.3389/fonc.2020.01787 (PMC7530237; doi:10.3389/fonc.2020.01787)
Supplement: Supplementary Table 1 — Sequences of PCR primer. The GenBank Accession number is shown: BTN3A1 (NM_001145), BIRC3 (NM_001165), CSF2RB (NM_000395), GIMAP7 (NM_153236), GZMB (NM_004131), HCLS1 (NM_005335), LCP2 (NM_005565), SELL (NM_000655), and GAPDH (NM_001256799). nt, nucleotide. [file Table_1.DOCX]

Supplementary Table S1 **|** Sequences of PCR primer.

| Name | | Sequence (5’ → 3’) | | Target position | |
| --- | --- | --- | --- | --- | --- |
| BTN3A1 |  | |  | |  |
| Forward  Reverse | AGGTGGAGGTAGGGGACAG  TCTGCACATTCTTACTGCACAC | | nt 1139-1157  nt 1198-1177 | |  |
| BIRC3 |  | |  | |  |
| Forward  Reverse | AAGCTACCTCTCAGCCTACTTT  CCACTGTTTTCTGTACCCGGA | | nt 317-338  nt 395-375 | |  |
| CSF2RB |  | |  | |  |
| Forward  Reverse | AGCGGCTTCAGGACTCTTG  CTGGGCATGAGGTGCTCTG | | nt 527-545  nt 614-596 | |  |
| GIMAP7  Forward  Reverse  GZMB  Forward  Reverse  HCLS1  Forward  Reverse  LCP2  Forward  Reverse  SELL  Forward  Reverse  GAPDH  Forward  Reverse | GCTCCCTGAGGATCGTTCTG  GCCCTGGAGTGTCTACAACAAG  CCCTGGGAAAACACTCACACA  GCACAACTCAATGGTACTGTCG  AGTGGGCCATGATGTGTCTG  CTCCCCATCGTTGCTCCTTT  GAGGAGCATCTTCACACGCAA  CGGCTCATAATCCGCGTCAT  ACCCAGAGGGACTTATGGAAC  GCAGAATCTTCTAGCCCTTTGC  GCACCGTCAAGGCTGAGAAC  TGGTGAAGACGCCAGTGGA | | nt 20-39  nt 199-178  nt 455-475  nt 564-543  nt 15-34  nt 118-99  nt 234-254  nt 441-422  nt 67-87  nt 210-189  nt 249-268  nt 386-368 | |  |

The GenBank Accession number is shown: BTN3A1 (NM_001145), BIRC3 (NM_001165), CSF2RB (NM_000395), GIMAP7 (NM_153236), GZMB (NM_004131), HCLS1 (NM_005335), LCP2 (NM_005565), SELL (NM_000655), and GAPDH (NM_001256799). nt, nucleotide.
