# Supplementary material for: Transcriptome-Based Network Analysis Unveils Eight Immune-Related Genes as Molecular Signatures in the Immunomodulatory Subtype of Triple-Negative Breast Cancer
Source: Front Oncol. 2020 Sep 18;10:1787. doi: 10.3389/fonc.2020.01787 (PMC7530237; doi:10.3389/fonc.2020.01787)
Supplement: Supplementary Table 2 — Number of genes in the 11 modules. [file Table_2.DOCX]

**Supplementary Table S2 |** Number of genes in the 11 modules.

| Modules | Gene numbers |
| --- | --- |
| Black | 180 |
| Blue | 558 |
| Brown | 543 |
| Green | 221 |
| Grey | 359 |
| Magenta | 91 |
| Pink | 109 |
| Purple | 69 |
| Red | 206 |
| Turquoise | 838 |
| Yellow | 426 |
