# Supplementary material for: Transcriptome-Based Network Analysis Unveils Eight Immune-Related Genes as Molecular Signatures in the Immunomodulatory Subtype of Triple-Negative Breast Cancer
Source: Front Oncol. 2020 Sep 18;10:1787. doi: 10.3389/fonc.2020.01787 (PMC7530237; doi:10.3389/fonc.2020.01787)
Supplement: Supplementary Table 3 — Top 50 hub genes. [file Table_3.docx]

**Supplementary Table S3 |** Top 50 hub genes.

| Symbol | q.Weighted | cor.Weighted | cor.Standard |
| --- | --- | --- | --- |
| FGL2 | 0 | -0.701363037 | -0.30762 |
| APOBEC3G | 0 | -0.70379213 | -0.26579 |
| IL2RB | 0 | -0.700357856 | -0.24916 |
| CD2 | 4.80E-13 | -0.686601691 | -2.46794 |
| BTN3A1 | 5.33E-13 | -0.684990784 | -0.29122 |
| LCP2 | 1.03E-12 | -0.677874166 | -0.24161 |
| TRIM22 | 3.80E-12 | -0.666395923 | -0.29463 |
| FAM26F | 1.37E-11 | -0.654239785 | -0.25071 |
| FGD2 | 1.37E-11 | -0.654224726 | -0.21563 |
| GZMB | 2.24E-11 | -0.648463233 | -0.2635 |
| CCL5 | 3.37E-11 | -0.643621766 | -0.22497 |
| CD53 | 7.57E-11 | -0.635848354 | -0.16404 |
| IL10RA | 1.07E-10 | -0.632165809 | -0.18067 |
| VEGFA | 1.61E-10 | 0.627825079 | 0.334256 |
| SAMHD1 | 2.14E-10 | -0.624647347 | -0.21157 |
| BTN3A2 | 2.68E-10 | -0.621398813 | -0.2487 |
| GIMAP6 | 2.68E-10 | -0.620870211 | -0.24858 |
| ERAP1 | 2.68E-10 | -0.620672447 | -0.30835 |
| SLA | 2.68E-10 | -0.620849763 | -0.17862 |
| IKZF1 | 3.62E-10 | -0.617362253 | -0.2082 |
| WIPF1 | 3.79E-10 | -0.616515317 | -0.22191 |
| NLRC5 | 4.07E-10 | -0.615447102 | -0.22887 |
| TRBC1 | 4.44E-10 | -0.61422503 | -0.19595 |
| PSMB10 | 1.43E-09 | -0.602234769 | -0.22222 |
| KDM5B | 1.92E-09 | 0.59889335 | 0.327009 |
| HCLS1 | 2.55E-09 | -0.595636815 | -0.1745 |
| RAC2 | 2.78E-09 | -0.594373554 | -0.15892 |
| SERPINB9 | 3.49E-09 | -0.591670058 | -0.21703 |
| TRAC | 3.95E-09 | -0.590019531 | -0.1922 |
| BIRC3 | 4.30E-09 | -0.588812595 | -0.19182 |
| SRGN | 4.59E-09 | -0.587799303 | -0.18343 |
| PSMB8 | 5.30E-09 | -0.58594455 | -0.26199 |
| CD48 | 5.50E-09 | -0.585264115 | -0.17417 |
| TNFSF13B | 6.89E-09 | -0.582528099 | -0.24438 |
| CD74 | 7.60E-09 | -0.581182576 | -0.17203 |
| UBE2L6 | 7.84E-09 | -0.58056201 | -0.26095 |
| LCK | 8.15E-09 | -0.579855335 | -0.19543 |
| SAMSN1 | 1.20E-08 | -0.575309254 | -0.20103 |
| CSF2RB | 1.27E-08 | -0.574426973 | -0.19194 |
| ARHGAP9 | 1.69E-08 | -0.571009069 | -0.15926 |
| HLA-DPA1 | 1.84E-08 | -0.569756575 | -0.20736 |
| HLA-DMB | 1.97E-08 | -0.568739673 | -0.16434 |
| PSMB9 | 2.21E-08 | -0.567190197 | -0.2339 |
| SELL | 2.23E-08 | -0.566707259 | -0.20006 |
| GIMAP7 | 2.23E-08 | -0.566569229 | -0.25643 |
| KRT19 | 2.40E-08 | 0.565508258 | 0.320649 |
| PLAC8 | 2.72E-08 | -0.563605366 | -0.21944 |
| CXCL9 | 4.17E-08 | -0.557776798 | -0.25714 |
| CORO1A | 4.17E-08 | -0.557770607 | -0.16553 |
| HLA-E | 4.52E-08 | -0.556604791 | -0.16273 |
